# BHLHE40 promotes osteoclastogenesis and abnormal bone resorption via c-Fos/NFATc1

**DOI:** 10.1186/s13578-022-00813-7

**Published:** 2022-05-26

**Authors:** Yufeng Zhang, Min Yang, Sheng Zhang, Zhiqiang Yang, Yufan Zhu, Yi Wang, Zhe Chen, Xuan Lv, Zan Huang, Yuanlong Xie, Lin Cai

**Affiliations:** 1 Department of Spine Surgery and Musculoskeletal Tumor, Department of Orthopedics, Zhongnan Hospital of Wuhan University, Wuhan, 430071 People’s Republic of China; 2 College of Life Sciences, Wuhan University, Wuhan, 430072 China

**Keywords:** BHLHE40, NFATc1, c-Fos, Osteoclast, Bone Resorption

## Abstract

**Background:**

Dysregulated osteoclast activity due to altered osteoclast differentiation causes multiple bone diseases. Osteoclasts are multinucleated giant cells derived from hematopoietic stem cells and play a major role in bone absorption. However, the mechanisms underlying the tight regulation of osteoclast differentiation in multiple pathophysiological status remain unknown.

**Results:**

We showed that Bhlhe40 upregulation is tightly associated with osteoclast differentiation and osteoporosis. Functionally, Bhlhe40 promoted osteoclast differentiation in vitro, and Bhlhe40 deficiency led to increased bone mass and decreased osteoclast differentiation in vivo. Moreover, Bhlhe40 deficient mice resisted estrogen deficiency and aging-induced osteoporosis. Mechanism study showed that the increase in bone mass due to Bhlhe40 deficiency was a cell intrinsic defect in osteoclast differentiation in these mice. BHLHE40 upregulated the gene expression of Fos and Nfatc1 by directly binding to their promoter regions. Notably, inhibition of Fos/Nfatc1 abrogated the enhanced osteoclast differentiation induced by BHLHE40 overexpression.

**Conclusions:**

Our research reveals a novel Bhlhe40/c-Fos/Nfatc1 axis involved in regulating osteoclastogenesis and shows that osteoporosis caused by estrogen deficiency and aging can be rescued by regulating Bhlhe40 in mice. This may help in the development of a new strategy for the treatment of osteoporosis.

**Supplementary Information:**

The online version contains supplementary material available at 10.1186/s13578-022-00813-7.

## Background

Bone homeostasis is dynamically maintained by balancing bone formation and bone resorption, which are mainly mediated by osteoblasts and osteoclasts (OCs), respectively. Unbalanced osteoblastogenesis and osteoclastogenesis cause multiple bone diseases. For instance, defective osteoclasts cause osteosclerosis and bone marrow failure [1–4]. Overactivated osteoclasts lead to bone loss and osteoporosis [5], and abnormal bone resorption is mostly caused by osteoclasts [6]. Regulating osteoclast activity is known to be a good method for the clinical treatment of abnormal bone resorption diseases. Indeed, various osteoclast drugs reduce abnormal bone resorption [7, 8]. However, these drugs also cause side effects including cardiovascular events and breast cancer risk [9]. In addition, these drugs usually fail to cure malignant osteoporosis and osteosclerosis [10, 11]. Although bone marrow transplantation is considered to be an effective method to treat malignant osteoporosis and osteosclerosis [12, 13], it is not available for most patients. There is an urgent need to study osteoclast differentiation to develop new strategies and identify potential targets.

OCs are multinucleated giant cells derived from hematopoietic stem cells (HSCs) and play a major role in bone absorption. Osteoclast differentiation is determined by macrophage colony stimulating factor (M-CSF) and receptor activator of NF-κB ligand (RANKL). The binding of RANKL and RANK recruits tumor necrosis factor receptor–associated factor 6 (TRAF6) and activates mitogen-activated protein kinase (MAPK) and NF-κB. MAPK ultimately stimulates the expression of nuclear factor of activated T cells (NFATc) [14] and activator protein-1 (AP-1) [15], which are two master transcription factors in osteoclast differentiation. Factors including inflammation, tumors, and estrogen deficiency may affect RANKL/RANK signaling and the expression of these transcription factors, leading to excessive activation of osteoclasts.

*Bhlhe40* (basic helix-loop-helix family member e40), also known as *Stra13*, *Sharp2* and *Dec1*, is a helix-loop-helix (BHLH) transcription factor [16, 17]. *Bhlhe40* is expressed in a variety of human cells [18], including osteoblasts, endothelial cells, monocytes and a selected population of resident macrophages [19]. It regulates target genes by binding to E-boxes or Sp1 sites in their promoter regions [16, 17]. *Bhlhe40* deficiency inhibits inflammation in periodontitis, obesity and hypertension [20–22]. *Bhlhe40* also regulates the polarization of macrophages by inhibiting p53-induced macrophage inhibitory cytokine-1 (*Mic1*) expression [23, 24]. In addition, *Bhlhe40* is an important immunomodulatory factor. Little is known about its role in osteoclast differentiation and bone absorption.

In this study, we found that *Bhlhe40* was upregulated during osteoclastogenesis and was associated with osteoporosis. *Bhlhe40*^*−/−*^ mice exhibited increased bone mass and resistance to bone loss induced by estrogen deficiency. *Bhlhe40* promoted osteoclastogenesis by upregulating *Fos/Nfatc1 *in vitro and in vivo. Our research reveals a novel *Bhlhe40/Fos/Nfatc1* axis involved in osteoclastogenesis and may provide a theoretical basis and potential targets for the development of novel strategies for bone resorption diseases.

## Results

### *Bhlhe40* upregulation correlated with OC differentiation and osteoporosis in mice

**Fig. 1:**
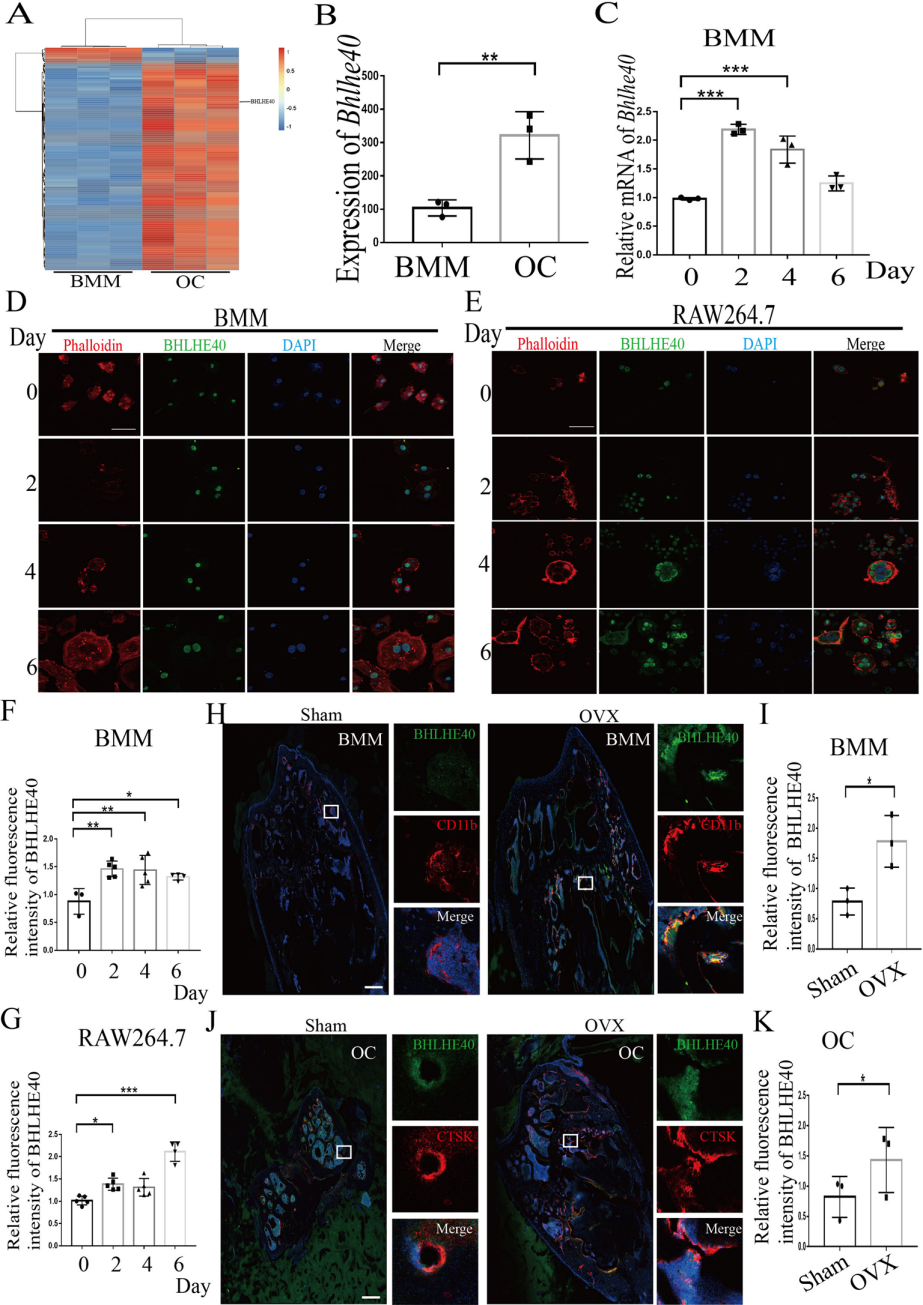
*Bhlhe40* upregulation correlates with OC differentiation and osteoporosis in mice. **A** Heatmap of mRNA in bone marrow macrophages (BMMs) and osteoclasts (OCs) from dataset GSE54779 on GEO. **B** Statistical of *Bhlhe40* expression between BMMs and OCs in Heatmap (n = 3). **C** RNA expression of *Bhlhe40* in BMMs during osteoclast differentiation (n = 3). **D** Immunofluorescence of BHLHE40 in BMMs during osteoclast differentiation (Scale bar, 50 μm). **E** Immunofluorescence of BHLHE40 in RAW264.7 during osteoclast differentiation (Scale bar, 50 μm). **F** Quantitative analysis (0D: n = 3; 6D: n = 4; Others: n = 5) of BHLHE40 in BMMs during osteoclast differentiation in **D**. **G** Quantitative analysis (6D: n = 4; Others: n = 5) of BHLHE40 in RAW264.7 during osteoclast differentiation in **E**. **H**, **I** Immunofluorescence and quantitative analysis (n = 3) of BHLHE40 (green) in BMMs between Sham and Osteoporosis mice. BMMs were stained with CD11b (red). Nucleus was stained with DAPI (blue) (Scale bar, 150 μm). **J**, **K** Immunofluorescence and quantitative analysis (n = 3) of BHLHE40 (green) in OCs between Sham and Osteoporosis mice. OCs were stained with CTSK (red). Nucleus was stained with DAPI (blue) (Scale bar, 150 μm). All data are mean ± SD; * P < 0.05, ** P < 0.01, *** P < 0.001. by unpaired (**B**, **C**, **F**, **G**), paired (**I**, **K**) Student’s t test and one-way ANOVA followed by Tukey’s post hoc test

To investigate the potential role of *Bhlhe40* in OC differentiation, we analyzed the GSE54779 dataset from the GEO database and found that the mRNA level of *Bhlhe40* was significantly upregulated in RANKL-treated mouse bone marrow macrophages (BMMs) during osteoclastogenesis (Fig. 1A, B). We further verified the upregulation of *Bhlhe40* mRNA in mouse BMMs undergoing osteoclast differentiation in the presence of M-CSF and RANKL (Fig. 1C and Additional file 1: Figure S1). Upregulation of BHLHE40 protein in BMMs and RAW264.7 cells was also observed by immunofluorescence during osteoclastogenesis (Fig. 1D–G). Moreover, we labeled BMMs or OCs with CD11b or CTSK and found *Bhlhe40* upregulation in BMMs and OCs from ovariectomized (OVX)-induced osteoporosis mice compared to those from sham-operated control mice (Fig. 1H–K). In addition, upregulation of *Bhlhe40* was also observed in elderly osteoporotic mice (Eld) (Additional file 2: Figure S2 A-D). These results suggested that BHLHE40 upregulation was tightly associated with OC differentiation and osteoporosis.

### *Bhlhe40* promoted OC differentiation in vitro

**Fig. 2:**
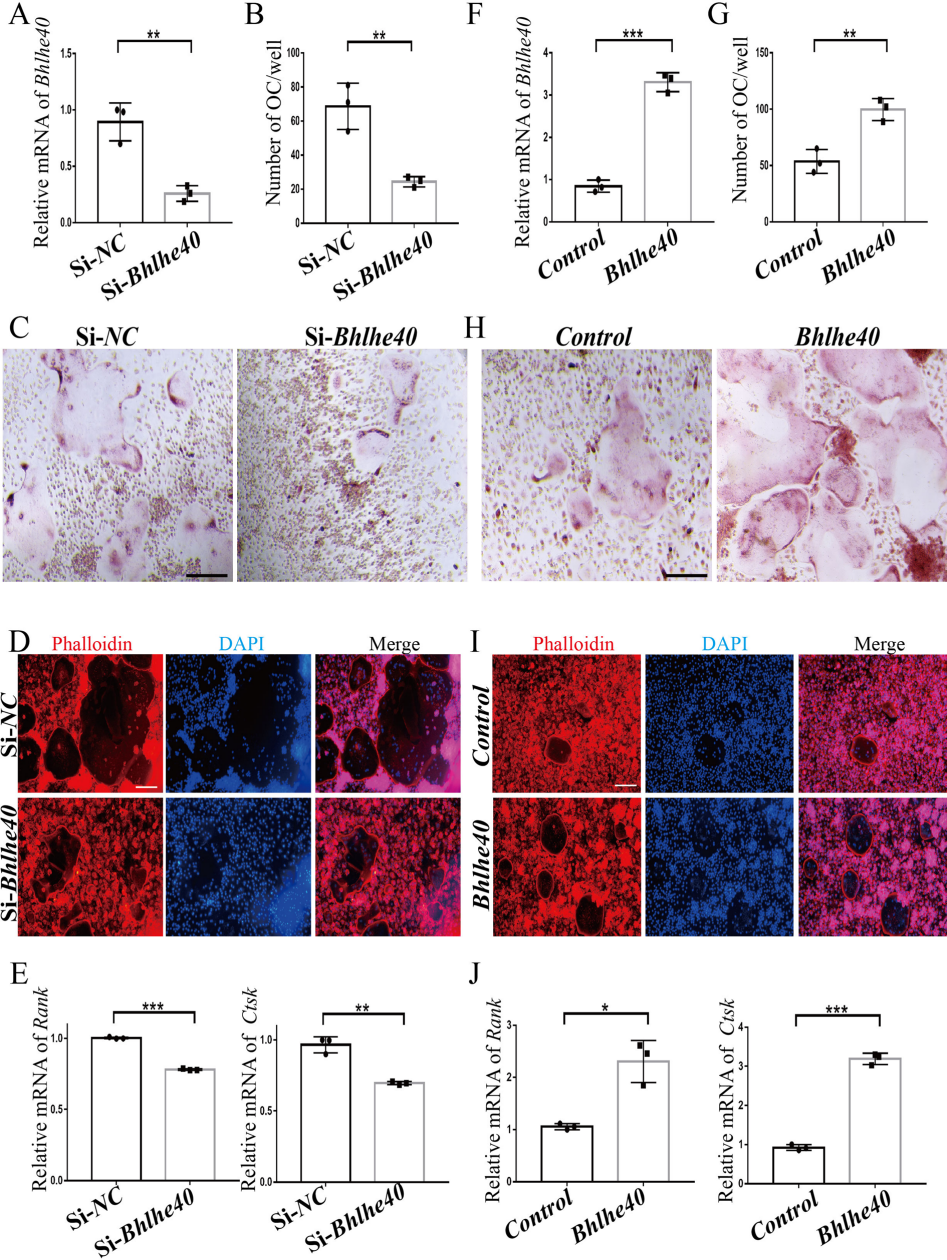
*Bhlhe40* promotes OC differentiation in vitro. **A** RNA expression of *Bhlhe40* in control (Si-*NC*) and *Bhlhe40* knockdown (Si-*Bhlhe40*) BMMs (n = 3). **B**, **C** Quantification (n = 3) and TRAP staining in Si-*NC* and Si-*Bhlhe40* (Scale bar, 50 μm). **D** Phalloidin staining in Si-*NC* and Si-*Bhlhe40* (Scale bar, 50 μm). **E** RNA expression of OC-specific genes in Si- *NC* and Si-*Bhlhe40* (n = 3). **F** RNA expression of *Bhlhe40* in control (*Control)* and *Bhlhe40* overexpressing (*Bhlhe40*) BMMs (n = 3). **G**, **H** Quantification (n = 3) and TRAP staining in *Control* and *Bhlhe40* (Scale bar, 50 μm). **I** Phalloidin staining in *Control* and *Bhlhe40* overexpression (Scale bar, 50 μm). **J** RNA expression of OC-specific genes in *Control* and *Bhlhe40* overexpression groups (n = 3). All data are mean ± SD; * P < 0.05, ** P < 0.01, *** P < 0.001. by unpaired Student’s t test

To investigate the potential role of *Bhlhe40* in osteoclastogenesis, we downregulated *Bhlhe40* by siRNA (Fig. 2A). *Bhlhe40* downregulation impaired the formation of osteoclasts (Fig. 2B–D) and the expression of OC-specific genes (Fig. 2E). In contrast, *Bhlhe40* overexpression induced the opposite phenotypes (Fig. 2F–J). In addition, *Bhlhe40* downregulation or upregulation did not seem to affect the viability of BMMs (Additional file 3: Figure S3). These results suggested that *Bhlhe40* may play a positive role in osteoclastogenesis.

### *Bhlhe40* deficiency impaired bone resorption in vivo

**Fig. 3:**
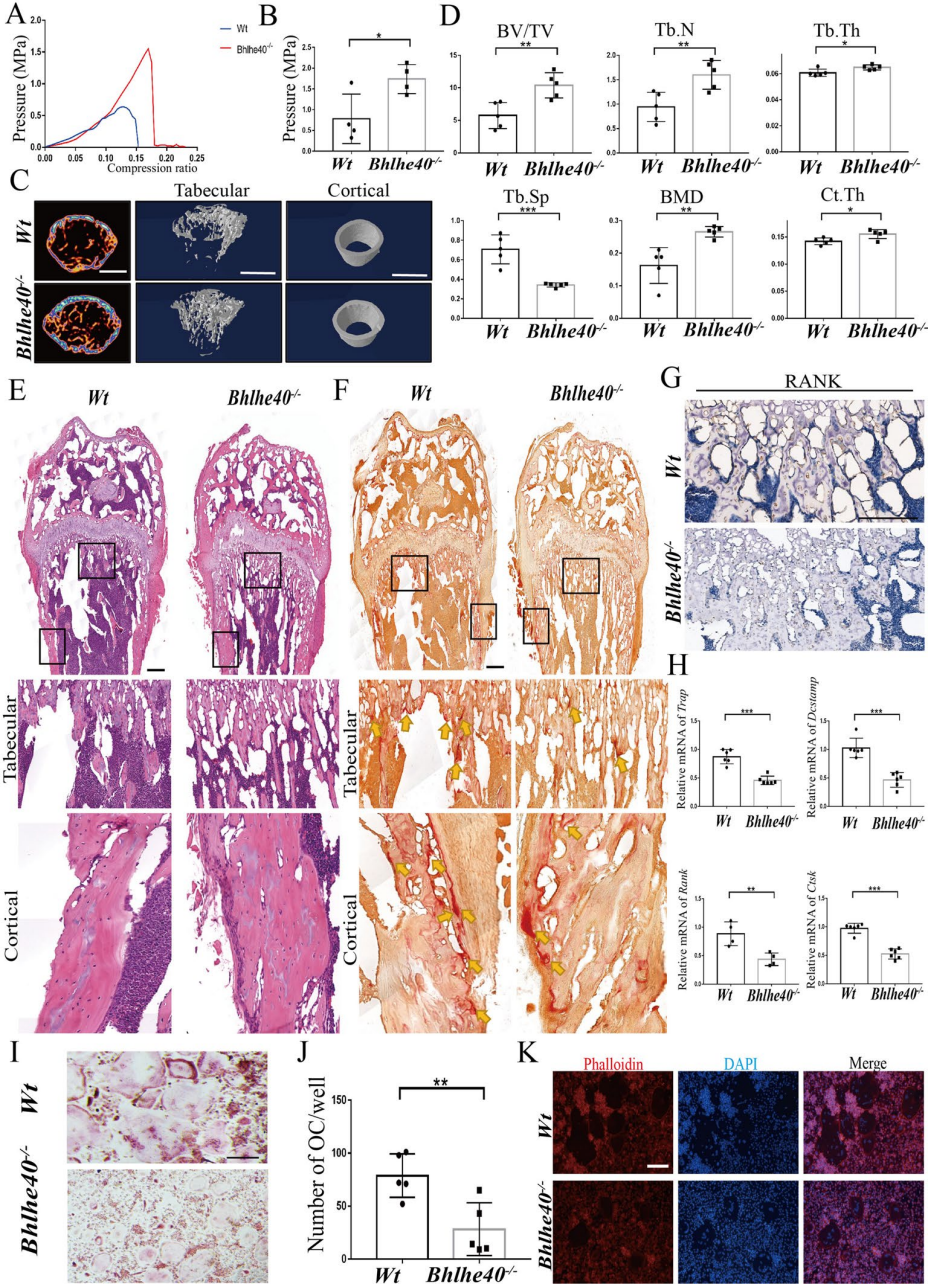
*Bhlhe40* deficiency impairs bone resorption in mice. **A**, **B** Mechanical strength and quantification of femurs between *Wt* and *Bhlhe40*^*−/−*^ mice at 4 weeks (n = 4). **C** Micro CT and three-dimensional (3D) reconstruction models of the femoral bone trabecula and cortical of *Wt* and *Bhlhe40*^*−/−*^ mice (4 weeks) (Scale bar, 1 mm). **D** Quantification of bone parameters by Micro CT from *Wt* and *Bhlhe40*^*−/−*^ mice (4 weeks) (n = 5). Bone volume fraction (BV/TV); Number of trabeculae (Tb. N); Trabecular bone thickness (Tb. Th); Trabecular bone separation (Tb. Sp); Bone mineral density (BMD); Cortical bone thickness (Ct. Th.) **E**, **F** H&E and TRAP staining of femur sections from *Wt* and *Bhlhe40*^*−/−*^ mice (4 weeks). The yellow arrowhead points to OCs (Scale bar, 150 um). **G** Immunohistochemistry of RANK in femurs from *Wt* and *Bhlhe40*^*−/−*^ mice at 4 weeks (Scale bar, 50 μm). **H** RNA expression of OC-specific genes in BMMs from *Wt* and *Bhlhe40*^*−/−*^ mice (RANK: n = 4; Others: n = 6). **I**, **J** TRAP staining and quantification (n = 5) of OCs from *Wt* and *Bhlhe40*^*−/−*^ mice (Scale bar, 50 μm). (K) Phalloidin staining of OCs from *Wt* and *Bhlhe40*^*−/−*^ mice (Scale bar, 50 μm). All data are mean ± SD; * P < 0.05, ** P < 0.01, *** P < 0.001. by unpaired Student’s t test

To further investigate the role of *Bhlhe40* in osteoclast formation in vivo, we took advantage of *Bhlhe40* knockout (*Bhlhe40*^*−/−*^) mice. *Bhlhe40* deletion was verified by PCR (Additional file 4: Figure S4). Femurs from 4-week-old *Wt* and *Bhlhe40*^*−/−*^ mice were separated to measure mechanical characteristics and bone mass. The mechanical strength of femurs from *Bhlhe40*^*−/−*^ mice was higher than that of femurs from *Wt* mice: they were able to endure more force before breaking than *Wt* mice (Fig. 3A, B). Quantitative microtomography (micro-CT) analysis showed that *Bhlhe40*^*−/−*^ mice exhibited lower values for trabecular spacing (Tb. Sp) as well as higher values for bone volume/tissue volume (BV/TV), number of trabeculae (Tb. N), trabecular thickness (Tb. Th), bone density (BMD) and cortical bone thickness (Ct. Th) than their *Wt* littermates (Fig. 3C, D). Consistently, H&E staining demonstrated an increased number of trabeculae and cortical thickness in *Bhlhe40*^*−/−*^ mice compared with *Wt* mice (Fig. 3E). TRAP staining showed that *Bhlhe40*^*−/−*^ mice had fewer osteoclasts (yellow arrow) on the bone surface than their *Wt* littermates (Fig. 3F). Furthermore, lower expression of RANK was observed in *Bhlhe40*^*−/−*^ mice than in their *Wt* littermates (Fig. 3G). In addition, BMMs isolated from 4-week-old *Bhlhe40*^*−/−*^ mice displayed less osteoclast differentiation, as evidenced by the reduced expression of OC specific genes (Fig. 3H) and decreased TRAP and phalloidin staining (Fig. 3I–K). We also isolated BMSCs from *Wt* and *Bhlhe40*^*−/−*^ mice and compared osteoblast differentiation. Although ALP activity appeared to be reduced in BMSCs from *Bhlhe40*^*−/−*^ mice, no significant difference in Alizarin Red S staining (ARS) or Von Kossa staining was observed between *Wt* and *Bhlhe40*^*−/−*^ mice (Additional file 5: Figure S5A). Less collagen was observed in the trabeculae of 4-week-old *Bhlhe40*^*−/−*^ mice than in those of *Wt* mice (Masson staining), whereas no significant difference in mineralization was observed (Goldner staining) (Additional file 5: Figure S5B, C). Notably, more ossification occurred in 3-day-old *Bhlhe40*^*−/−*^ mice than in their *Wt* littermates (Additional file 5: Figure S5D). Moreover, no significant difference in the number of mature osteoblasts expressing osteocalcin (OCN) was observed in *Bhlhe40*^*−/−*^ mice compared with their *Wt* littermates (Additional file 5: Figure S5E, F). These results suggested that *Bhlhe40* deficiency led to increased bone mass in vivo mainly due to impaired osteoclastogenesis rather than osteoblastogenesis.

### Osteosclerosis in *Bhlhe40*^*−/−*^ mice was dominated by osteoclasts instead of the microenvironment.

**Fig. 4:**
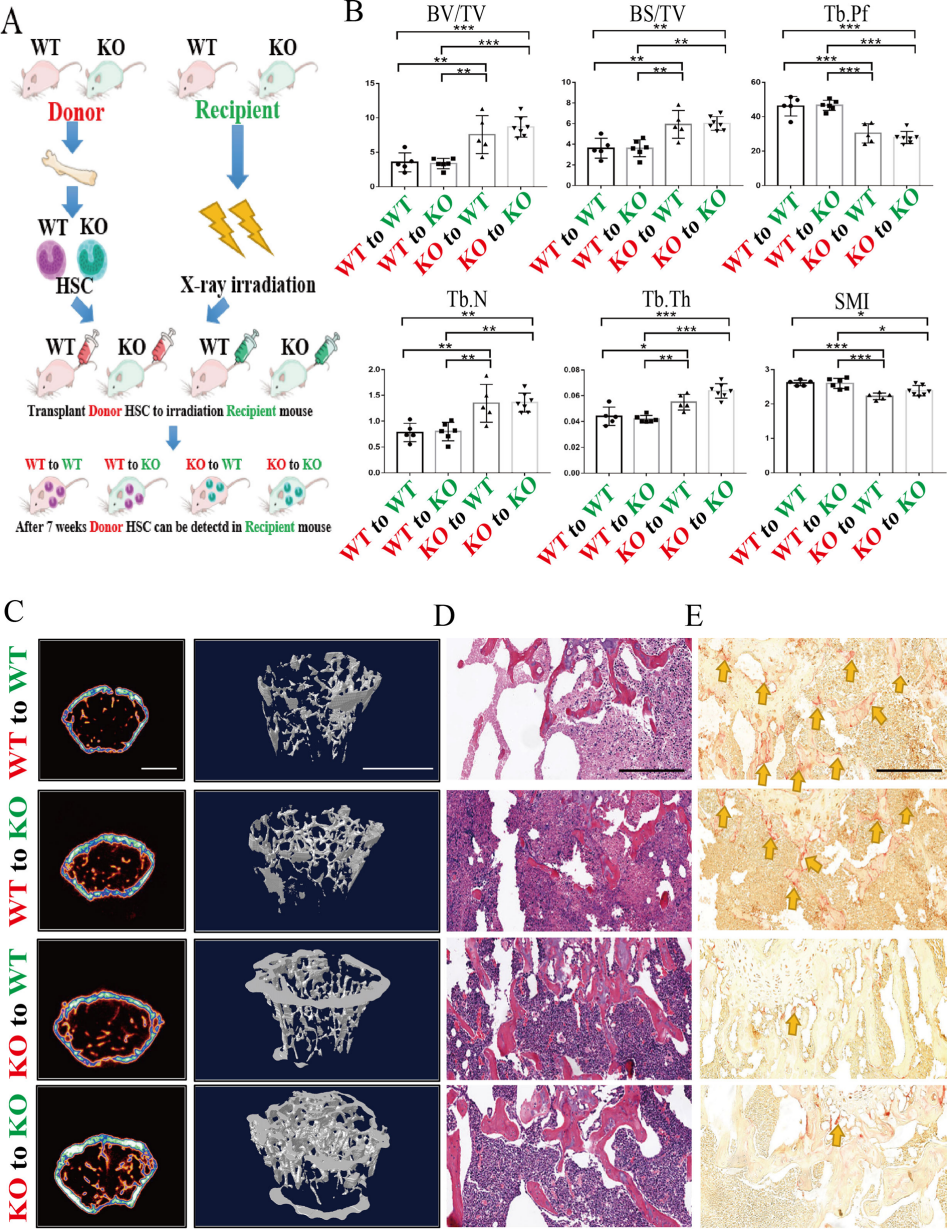
Osteosclerosis in *Bhlhe40*^*−/−*^ mice is dominated by osteoclasts instead of microenvironment. **A** Diagram of bone marrow transplantation. **B** Quantification of bone parameters by Micro CT from each group of mice for bone marrow transplantation (WT to WT: n = 5, WT to KO: n = 6, KO to WT: n = 5, KO to KO: n = 7). BV/TV; Bone surface area bone volume ratio (BS/TV); Structural model index (SMI); Tb. N; Tb. Th; Trabecular bone model factor (Tb. Pf). **C** Micro CT and 3D reconstruction models of the femoral trabecula from each group of mice for bone marrow transplantation (Scale bar, 1 mm). **D**, **E** H&E and TRAP staining of femurs from each group of mice for bone marrow transplantation. The yellow arrowhead points to OCs (Scale bar, 100 um). All data are mean ± SD; * P < 0.05, ** P < 0.01, *** P < 0.001. by one-way ANOVA followed by Tukey’s post hoc test

To verify that the increase in bone mass due to BHLHE40 deficiency was an intrinsic cell defect rather than other environmental factors, bone marrow transplantation was performed. Since adult osteoclasts are mainly derived from HSCs [6], this allows us to specifically study the effect of gene knockout in osteoclasts on bone mass, and excluding the influence of other components in the bone marrow. Bone marrow cells from *Wt* and *Bhlhe40*^*−/−*^ mice were engrafted into *Wt* and *Bhlhe40*^*−/−*^ recipient mice, respectively (Fig. 4A). Chimerism was verified by PCR (Additional file 6: Figure S6). We noticed that the phenotype of chimeric mice depended on the donor cells. For instance, the WT-to-WT group showed comparable values for bone mass, number of trabeculae, and number of osteoclasts compared to those of the WT-to-KO group. Consistently, the KO-to-WT group and KO-to-KO group exhibited comparable values for bone mass, number of trabeculae, and number of osteoclasts. Notably, chimeric mice receiving KO donor cells (the KO-to-WT and KO-to-KO groups) showed increased bone mass and number of trabeculae and a decreased number of osteoclasts compared with those of chimeric mice receiving *Wt* donor cells (Fig. 4B–E). Considering that the majority of osteoclasts are derived from hematopoietic cells, these observations demonstrated that the increase in bone mass in *Bhlhe40*^*−/−*^ mice was due to intrinsic defects in osteoclast differentiation.

### *Bhlhe40* directly regulated *c-Fos* and *Nfatc1* during osteoclast differentiation

**Fig. 5:**
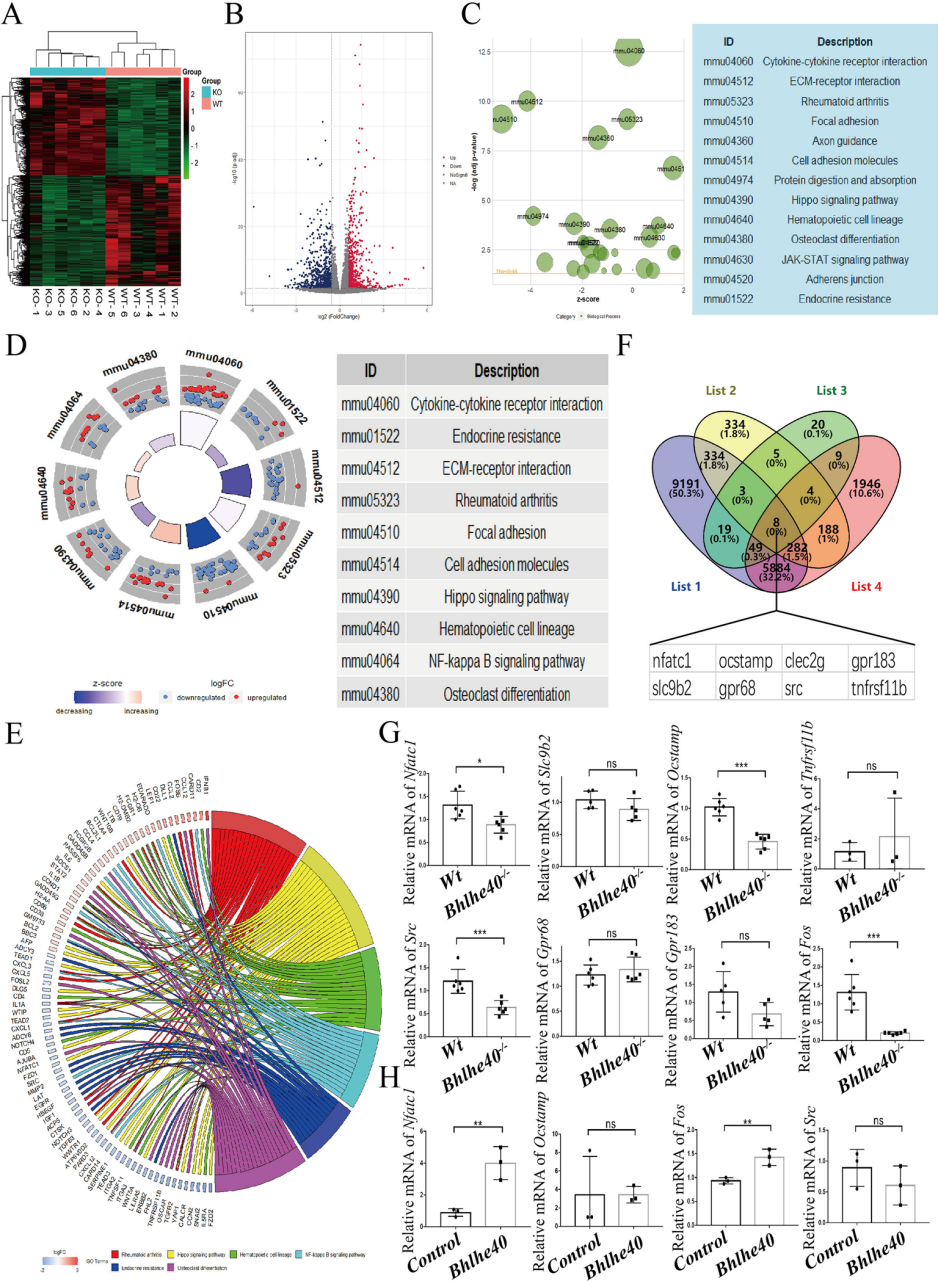
*Bhlhe40* regulates c-Fos and NFATc1 in osteoclast differentiation. **A** Heatmap of differentially expressed genes (DEGs) for BMMs in *Wt* versus *Bhlhe40*^*−/−*^ mice (n = 6). **B** Volcano plot of RNA-seq data of BMMs in *Wt* and *Bhlhe40*^*−/−*^ mice. **C** GOBubble plot of KEGG enrichment analysis in DEGs. **D** GOCircle plot of KEGG enrichment analysis of the DEGs. **E** GOchord of KEGG enrichment analysis of DEGs, including “Osteoclast differentiation”, “Rheumatoid arthritis”, “Hematopoietic cell lineage”, “Hippo signaling pathway”, “NF-κB signaling pathway”, and “Endocrine resistance”. **F** The predicted result of downstream genes regulated by *Bhlhe40*. List1 mentioned *Bhlhe40* target genes predicted by Gene Tranion Regulation Database. List2 mentioned the DEGs from our RNA-seq. List3 mentioned the key genes for osteoclastogenesis. List4 mentioned significant altered genes during osteoclastogenesis. **G** RNA expression of predicted genes in BMMs from *Wt* and *Bhlhe40*^*−/−*^ mice (*Nfatc1*: n = 6; *Fos*: n = 6; *Src*: n = 6; *Ocstamp*: n = 6; *Slc9b2*: n = 5; *Tnfrsf11b*: n = 3; *Grp68*: n = 6; *Grp183*: n = 5). **H** The mRNA expression levels of BHLHE40 target genes in Control and BHLHE40 overexpression BMMs (n = 3). All data are mean ± SD; ns P > 0.05, * P < 0.05, ** P < 0.01, *** P < 0.001. by unpaired Student’s t test

To dissect the mechanism by which *Bhlhe40* promotes osteoclast differentiation, we performed RNA-Seq. Heatmap and volcano analyses identified 1157 differentially expressed genes (DEGs) between *Wt* and *Bhlhe40*^*−/−*^ mice, of which 541 genes were upregulated and 616 genes were downregulated (Fig. 5A, B). KEGG analysis of DEGs revealed a significant enrichment of genes related to cytokine-cytokine receptor interactions, endocrine resistance, osteoclast differentiation and the NF-κB signaling pathway (Fig. 5C, D). The enrichment of genes related to osteoclasts in vivo was further analyzed by GOChord. The enriched genes were assigned to the following categories: “osteoclast differentiation”, “NF-κB signaling pathway”, “rheumatoid arthritis”, “Hippo signaling pathway”, “endocrine resistance”, and “hematopoietic cell lineage” (Fig. 5E). These observations suggest that *Bhlhe40* may regulate the expression of osteoclast differentiation genes. We performed Venn diagram analysis by using the BHLHE40 target genes predicted by the Gene Transcription Regulation Database (list 1), the DEGs from our RNA-seq (list 2), key genes involved in osteoclast differentiation (list 3) and osteoclast differentiation-regulated DEGs from GEO databases (list 4) and 8 genes were identified as common genes including *Nfatc1* (Fig. 5F). The expression changes in *Wt* and *Bhlhe40*^*−/−*^ mice were verified for seven genes (*Clec2g* could not be detected by qPCR due to its low abundance in cells) (Fig. 5G). However, only 4 genes were further confirmed in Bhlhe40 overexpressing cells, and no significant changes in *Src* and *Ocstamp* expression were observed (Fig. 5H). Although *Fos* was not identified as a DEG in RNA-Seq, we verified its downregulation in *Bhlhe40*^*−/−*^ mice and its upregulation in *Bhlhe40* overexpressing cells (Fig. 5G, H). Taken together, these results suggested that *Bhlhe40* may promote osteoclastogenesis by upregulating the master regulators *Fos* and *Nfatc1*.

### Bhlhe40 directly targeted c-Fos and Nfatc1

**Fig. 6:**
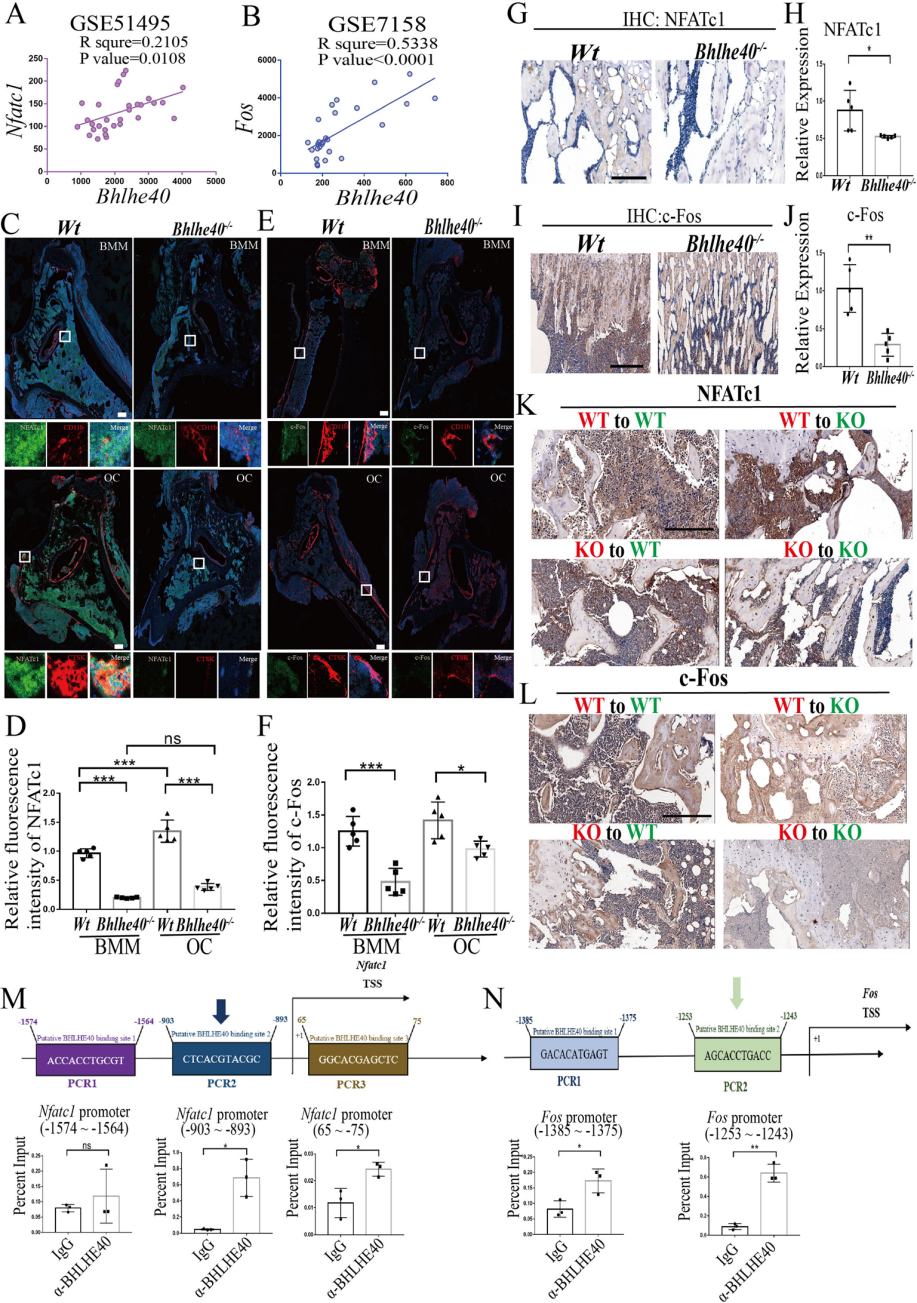
*Bhlhe40* deficiency leads to a decrease in *c-Fos*/*Nfatc1* and directly targets *Fos* and *Nfatc1*. **A** The correlation expression of *Bhlhe40* and *Nfatc1* (GSE51495 dataset) (n = 30). **B** The correlation expression of *Bhlhe40* and *Fos* (GSE7158 dataset) (n = 26). **C**, **D** Immunofluorescence and quantitative (n = 5) of NFATc1 (green) in BMMs or OCs between *Wt* and *Bhlhe40*^*−/−*^ mice. BMMs were stained with CD11b (red). OCs were stained with CTSK (red). Nucleus was stained with DAPI (blue) (Scale bar, 150 μm). **E**, **F** Immunofluorescence and quantitative (n = 5) of c-Fos (green) in BMMs or OCs between *Wt* and *Bhlhe40*^*−/−*^ mice. BMMs were stained with CD11b (red). OCs were stained with CTSK (red). Nucleus was stained with DAPI (blue) (Scale bar, 150 μm). **G**, **H** Immunohistochemistry of NFATc1 and quantitative analysis (n = 5) in femurs from *Wt* and *Bhlhe40*^*−/−*^ mice (Scale bar, 50 μm). **I**, **J** Immunohistochemistry of c-Fos and quantitative analysis (n = 5) in femurs from *Wt* and *Bhlhe40*^*−/−*^ mice (Scale bar, 50 μm). **K** Immunohistochemistry of NFATc1 in femurs from each group of mice for bone marrow transplantation (Scale bar, 50 μm). **L** Immunohistochemistry of c-Fos in femurs from each group of mice for bone marrow transplantation (Scale bar, 50 μm). **M** Anti-BHLHE40 ChIP assay for the *Nfatc1* promoter in BMMs. Predicted anti-BHLHE40 binding sites are indicated. Immunoprecipitated DNA was amplified by qPCR (n = 3). Normal rabbit IgG was used as a negative control. Results are presented as ChIP/Input. **N** Anti-BHLHE40 ChIP assay for the *Fos* promoter. Predicted Anti-BHLHE40 binding sites are indicated. Immunoprecipitated DNA was amplified by qPCR (n = 3). Normal rabbit IgG was used as a negative control. Results are presented as ChIP/Input. All data are mean ± SD; ns P > 0.05, * P < 0.05, ** P < 0.01, *** P < 0.001. by unpaired Student’s t test, two-tailed and one-way ANOVA followed by Tukey’s post hoc test

To further investigate the regulatory role of *Bhlhe40* in *Nfatc1* and *Fos* expression, we first confirmed the correlation between *BHLHE40* and *NFATC1* expression (GSE51496) or *FOS* (GSE7158) in the GEO database (Fig. 6A, B). Immunofluorescence showed that *Bhlhe40*^*−/−*^ mice (4 weeks old) expressed less NFATc1 and c-Fos in both BMMs (CD11b +) and OCs (CTSK +) than their *Wt* littermates (Fig. 6C–F). Downregulation of NFATc1 and c-Fos in *Bhlhe40*^*−/−*^ mice was also detected by immunohistochemistry (Fig. 6G–J). Consistently, mice receiving *Wt* donor cells had higher expression of NFATc1 and c-Fos than mice receiving *Bhlhe40*^*−/−*^ donor cells (Fig. 6K, L). These observations suggested that Bhlhe40 regulated *Nfatc1* and *Fos* during osteoclast differentiation. To explore whether BHLHE40 directly regulates *Fos* and *Nfatc1*, 3 putative Bhlhe40-binding sites were identified in the *Nfatc1* gene promoter region, and 2 sites in the *Fos* gene promoter region were identified (Fig. 6M, N) by JASPAR (http://jaspar.genereg.net). ChIP analysis in BMMs demonstrated that the amount of DNA in these putative binding sites was significantly increased by the BHLHE40 antibody compared to the preimmune control antibody (Fig. 6M, N). These findings demonstrated that BHLHE40 directly bound to the promoter regions of the *Nfatc1* and *Fos* genes and regulated their expression.

### *c-Fos*/*Nfatc1* was required for* Bhlhe40* function during osteoclast differentiation

**Fig. 7:**
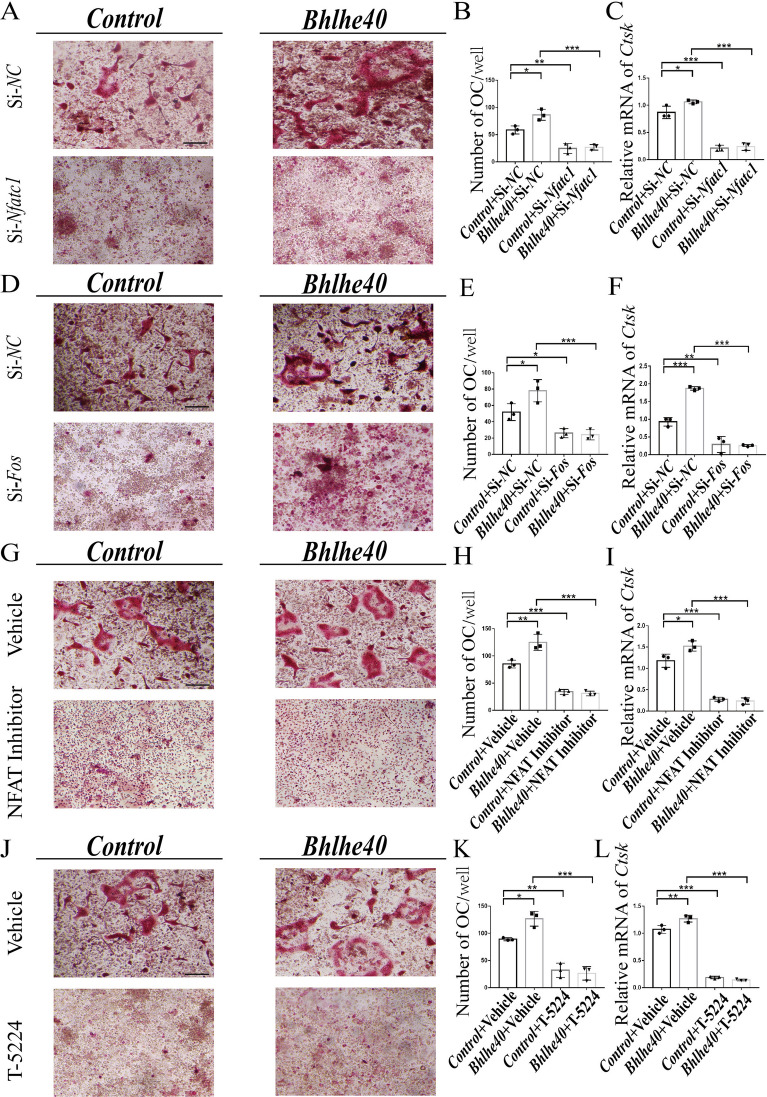
*c-Fos*/*Nfatc1* is required for *Bhlhe40* function in osteoclast differentiation. **A**, **B** TRAP staining and quantification (n = 3) to detect osteoclast differentiation of BMMs in *Control*, *Bhlhe40* overexpression, *Control* treated with Si-*Nfatc1*, and *Bhlhe40* overexpression treated with Si-*Nfatc1* groups (Scale bar, 50 μm). **C** OC-specific gene expression of BMMs in *Control*, *Bhlhe40* overexpression, *Control* treated with Si-*Nfatc1*, and *Bhlhe40* overexpression treated with Si-*Nfatc1* groups (n = 3). **D**, **E** TRAP staining and quantification (n = 3) to detect osteoclast differentiation of BMMs in *Control*, *Bhlhe40* overexpression, *Control* treated with Si-*Fos*, and *Bhlhe40* overexpression treated with Si-*Fos* groups (Scale bar, 50 μm). **F** OC-specific gene expression of BMMs in *Control*, *Bhlhe40* overexpression, *Control* treated with Si-*Fos*, and *Bhlhe40* overexpression treated with Si-*Fos* groups (n = 3). **G**, **H** TRAP staining and quantification (n = 3) to detect osteoclast differentiation of BMMs in *Control*, *Bhlhe40* overexpression, *Control* treated with NFAT Inhibitor, and *Bhlhe40* overexpression treated with NFAT Inhibitor groups (Scale bar, 50 μm). **I** OC-specific gene expression of BMMs in *Control*, *Bhlhe40* overexpression, *Control* treated with NFAT Inhibitor, and *Bhlhe40* overexpression treated with NFAT Inhibitor groups (n = 3). NFAT Inhibitor reduced the expression of NFATc1. **J**, **K** TRAP staining and quantification (n = 3) to detect osteoclast differentiation of BMMs in *Control*, *Bhlhe40* overexpression, *Control* treated with T-5224, and *Bhlhe40* overexpression treated with T-5224 groups (Scale bar, 50 μm). **L** OC-specific genes expression of BMMs in *Control*, *Bhlhe40* overexpression, *Control* treated with T-5224, and *Bhlhe40* overexpression treated with T-5224 groups (n = 3). T-5224 reduced the expression of c-Fos. All data are mean ± SD; * P < 0.05, ** P < 0.01, *** P < 0.001. by one-way ANOVA followed by Tukey’s post hoc test

To verify the role of *Nfatc1* and *Fos* in *Bhlhe40*-mediated osteoclastogenesis, *Nfatc1* and *Fos* were knocked down by siRNA (Additional file 7: Figure S7) in BHLHE40 overexpressing cells. *Nfatc1* knockdown or *Fos* knockdown abrogated the enhanced osteoclastogenesis induced by *Bhlhe40* overexpression, as evidenced by a decrease in TRAP + cells and osteoclast-specific gene expression (Fig. 7A–F). Consistently, an NFATc1 inhibitor (NFAT Inhibitor) or a c-Fos inhibitor (T-5224) also generated similar results (Fig. 7G–L). These results suggested that *Nfatc1* and *Fos* were required for Bhlhe40 function in osteoclast differentiation.

### BHLHE40 deficiency alleviated OVX and aging-induced osteoporosis in vivo

**Fig. 8:**
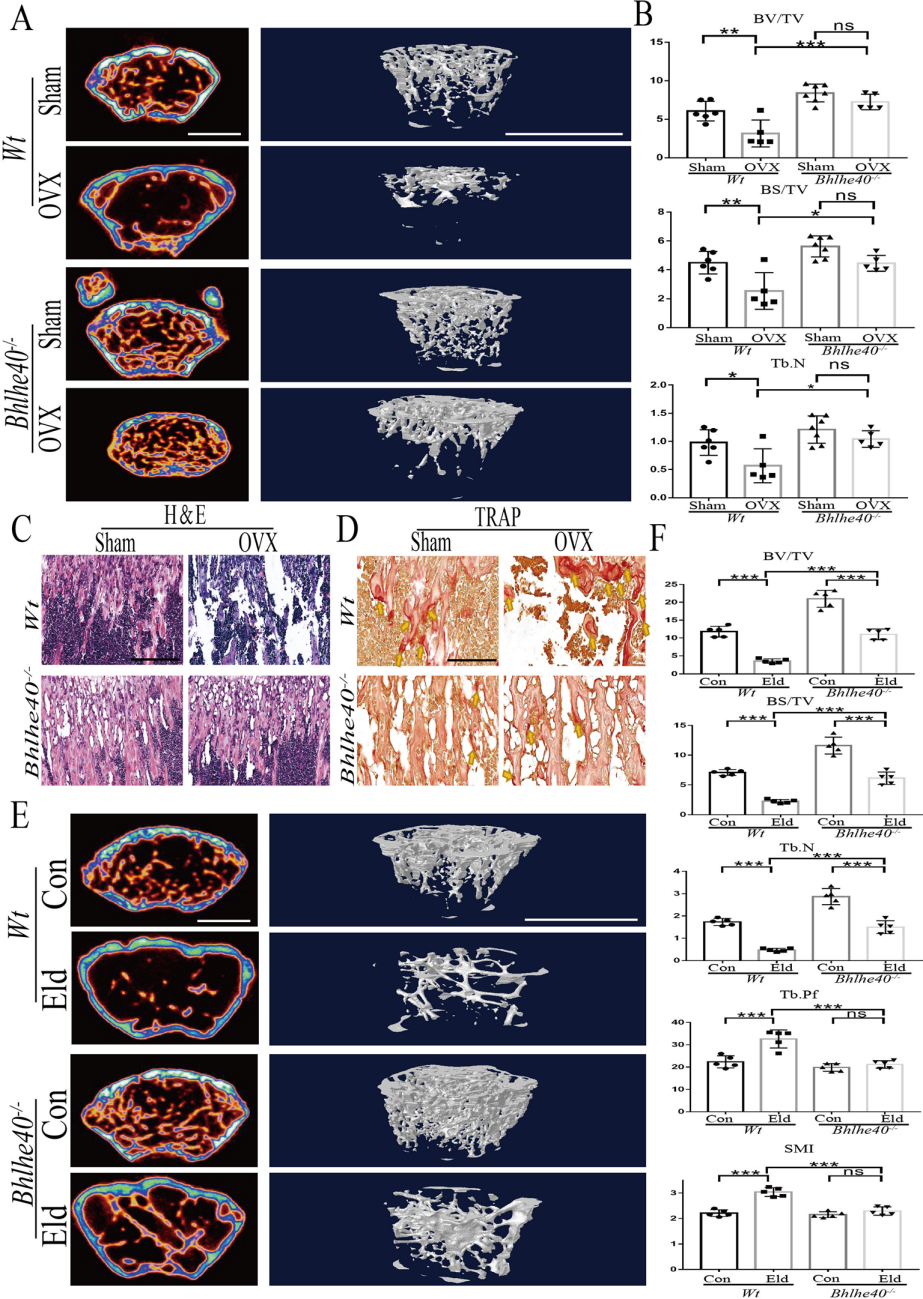
BHLHE40 deficiency alleviates bone loss due to estrogen deficiency in vivo. **A** Representative Micro CT images of femurs from *Wt* (Sham) *Wt* (OVX)*, Bhlhe40*^*−/−*^ (Sham) and *Bhlhe40*^*−/−*^ (OVX) mice (Scale bars, 1 mm). **B** Quantification of cortical bone parameters by Micro CT from *Wt* (Sham) *Wt* (OVX)*, Bhlhe40*^*−/−*^ (Sham) and *Bhlhe40*^*−/−*^ (OVX) mice. BV/TV; BS/TV; Tb. N (*Wt* (Sham): n = 6, *Wt* (OVX): n = 5 *Bhlhe40*^*−/−*^ (Sham): n = 7, *Bhlhe40*^*−/−*^ (OVX): n = 5). **C** H&E staining of femur sections from *Wt* (Sham) *Wt* (OVX)*, Bhlhe40*^*−/−*^ (Sham) and *Bhlhe40*^*−/−*^ (OVX) mice (Scale bar, 200 μm). **D** TRAP staining of femur sections from *Wt* (Sham) *Wt* (OVX)*, Bhlhe40*^*−/−*^ (Sham) and *Bhlhe40*^*−/−*^ (OVX) mice. The yellow arrowhead points to OCs (Scale bar, 100 μm). **E** Representative Micro CT images of femurs from *Wt* (2 months) *Wt* (12 months), *Bhlhe40*^*−/−*^ (2 months) and *Bhlhe40*^*−/−*^ (12 months) mice (Scale bars, 1 mm). **F** Quantification (n = 5) of cortical bone parameters by Micro CT from *Wt* (2 months), *Wt* (12 months), *Bhlhe40*^*−/−*^ (2 months) and *Bhlhe40*^*−/−*^ (12 months) mice. BV/TV; BS/TV; Tb. N; Tb.Pf; SMI. All data are mean ± SD; * P < 0.05, ** P < 0.01, *** P < 0.001. by one-way ANOVA followed by Tukey’s post hoc test

To examine whether BHLHE40 played a role in osteoporosis, an OVX-induced osteoporosis mouse model was established. Micro-CT results showed that OVX *Wt* mice exhibited significant bone mass loss and decreased values for BV/TV, bone surface area/tissue volume (BS/TV), and Tb. N compared with those of the Sham *Wt* group (Fig. 8A, B). In sharp contrast, OVX did not cause significant bone mass loss or significant changes in these values in *Bhlhe40*^*−/−*^ mice (Fig. 8A, B). Similarly, H&E staining showed that OVX did not significantly change bone mass or Tb. N in *Bhlhe40*^*−/−*^ mice but did so in *Wt* mice (Fig. 8C). There were more TRAP-positive cells (yellow arrow) in OVX *Wt* mice than in Sham *Wt* mice whereas no significant changes were observed between OVX and Sham *Bhlhe40*^*−/−*^ mice (Fig. 8D). Moreover, micro-CT results indicated that the senile *Wt* mice showed significant bone loss and decreased BV/TV, BS/TV and Tb. N values compared with those of the adult *Wt* group (bone mass decreased by approximately 75%), whereas *Bhlhe40* deficiency partially rescued bone loss in senile mice (bone mass decreased by approximately 40%) (Fig. 8E, F). Furthermore, the values of indicators of osteoporosis (trabecular bone model factor (Tb. Pf) and structural model index (SMI)) increased in senile *Wt* mice, while aging did not cause similar changes in *Bhlhe40*^*−/−*^ mice (Fig. 8F). These findings suggested that BHLHE40 may play an important role in osteoporosis, especially OVX-induced osteoporosis.

## Discussion

Osteoclasts are critical for bone resorption and abnormal activation of osteoclasts causes a variety of bone diseases including osteoporosis. OC differentiation is controlled by the combined action of multiple transcription factors [25]. *Bhlhe40* is a helix-loop-helix transcription factor that plays a key role in various biological processes and is expressed in a variety of human tissues and cells, including resident macrophages [18, 19]. However, the role of Bhlhe40 in osteoclastogenesis and its therapeutic effect on abnormal bone resorption-related diseases are still unclear. Here, we demonstrated that *Bhlhe40* upregulation was associated with abnormal bone resorption diseases and that *Bhlhe40* deficiency inhibited osteoclastogenesis in vitro and in vivo. In addition, bone marrow transplantation showed that osteoclasts were the culprit of osteosclerosis caused by Bhlhe40 deficiency. Through RNA-seq and chromatin immunoprecipitation, we identified a new *Bhlhe40*/*Fos*/*Nfatc1* axis through which *Bhlhe40* affects osteoclastogenesis. Finally, we established a mouse model of estrogen-deficient and senile osteoporosis and found a significant improvement in abnormal bone resorption in *Bhlhe40*-deficient mice, suggesting that *Bhlhe40* is an effective target for the treatment of osteoporosis.

Although limited studies have shown that *Bhlhe40* may promote PI3KCA/Akt/GSK3β-regulated osteoblastogenesis [26], the effect of *Bhlhe40* on osteoclasts has not been proven, and maintaining bone mass balance is a complex process that requires the participation of osteoblasts and osteoclasts. In contrast, the lack of Bhlhe40 reduces cement loss caused by Porphyromonas gingivalis in a periodontitis model [20]. These studies seem to be contradictory and prompted us to further explore how Bhlhe40 regulates bone mass in abnormal bone resorption diseases. To this end, we isolated bone marrow mesenchymal cells from 4-week-old *Wt* and *Bhlhe40*^*−/−*^ mice for verification. Interestingly, *Bhlhe40* knockout affected the number of ALP + cells but did not affect mineralization, as evidenced by comparable ARS staining at 21 days and Von Kossa staining at 28 days (Additional file 5: Figure S5A). Masson and Goldner staining of the femurs in *Wt* and *Bhlhe40*^*−/−*^ mice may support this possibility: Collagen reduction was observed whereas mineralization showed no significant change in *Bhlhe40*^*−/−*^ mice. OCN is characteristic of mature osteoblasts and is considered an indicator of mineralization [27]. We failed to find a significant difference in the number of OCN + cells by immunohistochemistry. These observations exclude the possibility that the increased bone mass in *Bhlhe40*^*−/−*^ mice is due to increased osteoblastogenesis. Indeed, bone marrow transplantation demonstrated that the phenotype of *Wt* or *Bhlhe40*^*−/−*^ recipient mice depended on the donor cells (Fig. 4). Many studies have shown that osteoclasts develop through the fusion of monocyte precursors derived from HSCs in the presence of M-CSF and RANKL [28, 29]. In contrast, it is difficult for osteoblasts or bone cells to colonize the bone marrow through bone marrow transplantation [30]. If the bone mass changes in *Bhlhe40*^*−/−*^ mice are dominated by osteoblasts or other bone cells, the phenotype of the recipient mice should depend on themselves rather than the donors. Therefore, our experiment proved that the bone mass changes in *Bhlhe40*^*−/−*^ mice are dominated by osteoclasts. Taken together, our observations indicate that the changes in bone mass caused by *Bhlhe40* deficiency are most likely dominated by osteoclasts rather than osteoblasts.

Our RNA-seq and subsequent experiments have shown that *Bhlhe40* affects osteoclastogenesis by regulating the transcription of *Nfatc1* and *Fos*. The *Fos*/*Nfatc1* pathway plays a crucial role in the formation of osteoclasts. NFATc1 promotes the expression of a variety of osteoclast-specific genes [31]. c-Fos is an important part of transcriptional AP-1. BMMs lacking c-Fos cannot differentiate into osteoclasts, and mice lacking c-Fos develop osteosclerosis, indicating the key role of c-Fos in osteoclastogenesis [32, 33]. Indeed, downregulation and inhibition of *Fos* or *Nfatc1* significantly reversed the increase in osteoclastogenesis in *Bhlhe40* overexpressing BMMs, which indicates that *Bhlhe40*-mediated regulation of osteoclastogenesis depends on *Fos*/*Nfatc1*. Our ChIP-PCR confirmed the direct binding of BHLHE40 to the promoter regions of the *Nfatc1* and *Fos* genes. In addition, many studies have demonstrated that *Bhlhe40* promotes inflammation [20, 34, 35], which causes bone loss by promoting osteoclast differentiation [36, 37]. Our RNA-Seq also revealed NF-κB signaling and rheumatoid arthritis, which are tightly associated with inflammation. We do not exclude the possibility that *Bhlhe40* promotes osteoclast differentiation by enhancing inflammation.

Many osteolytic diseases are caused by the excessive activation of OCs. As mentioned earlier, abnormal bone resorption is mainly inhibited clinically by regulating the function of OCs. However, most drugs have off-target effects and potential side effects [38, 39]. In addition, these drugs usually cannot cure malignant osteoporosis and osteopetrosis [10, 11]. Studies have shown that some refractory osteoporosis in humans and mice can be partially treated by bone marrow transplantation [40, 41], but this approach is not available to most patients and causes substantial pain. Here, our research showed that *Bhlhe40* deficiency can prevent excessive bone resorption. The bone mass and trabecular bone number of ovariectomized *Bhlhe40*^*−/−*^ mice were similar to those of the Sham group, while the bone mass and trabecular bone number of ovariectomized *Wt* mice were significantly decreased. On the other hand, because aging is a process involving an overall decline in body functions, *Bhlhe40* deficiency cannot completely reverse the effects of aging, but it still demonstrated significant therapeutic effects in senile models. In addition, senile *Bhlhe40*^*−/−*^ mice showed similar bone mass as adult *Wt* mice, which demonstrated the possibility of using *Bhlhe40* as a treatment target for aging-induced osteoporosis. These results indicate that diseases related to abnormal bone resorption can be cured by downregulating *Bhlhe40*. In addition, *Tcirg1*, considered to be the main case of malignant infantile osteopetrosis (MIOP) [42], was observed to be downregulated in *Bhlhe40*^*−/−*^ mice by RNA-Seq. This result reveals the possibility of using *Bhlhe40* as a potential therapeutic target for osteopetrosis. Since the lack of *Bhlhe40* also partially affects osteogenesis, the inhibition of *Bhlhe40* may have broad application prospects in OVX and inflammation-related osteolysis, which are believed to be dominated by osteoclasts. In senile osteoporosis, which is caused by multiple factors [43], the inhibition of *Bhlhe40* should be combined with other treatments.

## Conclusion

In summary, our research demonstrated that *Bhlhe40* may regulate OC differentiation through *Fos/Nfatc1* signaling. Bone mass and trabecular bone are important determinants of bone strength in humans. Loss of bone mass in osteoporosis can affect bone strength and may cause fractures. Our research showed that osteoporosis caused by estrogen deficiency and aging can be rescued by regulating Bhlhe40. Therefore, *Bhlhe40* may be a promising target for the treatment of osteoporosis.

## Methods

### Cell culture and osteoclast differentiation

The mouse macrophage cell line RAW264.7 (ATCC, Cat# TIB-71™) and human kidney epithelial cell line 293T (ATCC, Cat# CRL-3216™) were cultured in DMEM (HyClone, Cat# SH30243.01B) supplemented with 10% fetal bovine serum (FBS) at 37 °C in a 5% CO_2_ atmosphere. All cell lines were routinely screened for mycoplasma contamination using a Mycoplasma Stain Assay Kit (Beyotime, Cat# C0296).

Bone marrow macrophages (BMMs) were isolated from 4-week-old male C57BL/6J mice as previously described [44, 45]. In short, the femur was separated under aseptic conditions, and the bone marrow cavity was flushed with α-MEM (HyClone, Cat# SH30265.01B) containing 10% FBS and 1% penicillin–streptomycin. The cell suspension was cultured in complete α-MEM at 37 °C in a 5% CO_2_ atmosphere overnight to filter off adherent mesenchymal stem cells. The supernatant was transferred to a new cell culture flask, M-CSF was added to a final concentration of 50 ng/ml, and the cells were cultured for another 3 days to allow BMMs to adhere and proliferate.

For OC differentiation, cells were cultured in complete α-MEM containing 30 ng/ml M-CSF (R&D Systems, Cat# 416-ML-010) and 50 ng/ml RANKL (R&D Systems, Cat# 462-TEC-010) for 6 days. The medium was changed every other day. Multinucleated osteoclasts were fixed in 4% paraformaldehyde at room temperature for 20 min, soaked in PBS containing 0.5% Triton-X for 30 min, and stained with tartrate-resistant acid phosphatase (TRAP) solution (Servicebio, Cat# G1050) at 37 °C for 50 min. Osteoclast differentiation was also confirmed by immunofluorescence staining with DAPI (Servicebio, Cat# G1012) and phalloidin (Servicebio, Cat# G1041). Fluorescent images were acquired using an inverted microscope system (Olympus, IX73, Japan). Cells with 3 or more nuclei were counted. Statistics are based on the number of OCs in 8 fields. For drug experiments, 10 µM NFAT Inhibitor (MCE, Cat# HY-P1026) was used to inhibit NFATc1, and 10 µM T-5224 (MCE, Cat# HY-12270) was used to inhibit c-Fos.

### Isolation, culture and differentiation of bone mesenchymal stem cells (BMSCs)

Primary BMSCs were collected from 4-week-old Wt and Bhlhe40-/- male mice as previously described [46]. In brief, the mechanically separated femoral and tibial diaphysis were digested with α-MEM containing 1 mg/ml collagenase II (Gibco, Cat# 17101015) and 2 mg/ml 100 mg dispase II (Millipore Sigma, Cat# D4693) for 2 h. Cells were cultured in complete α-MEM at 37 °C in a 5% CO2 atmosphere. The 3rd to 5th generation BMSCs were used in this study.

For osteoblast differentiation, cells were cultured in α-MEM containing 10% FBS, 10 nM dexamethasone (Sigma-Aldrich, Cat # D2915), 10 mM β-glycerophosphate (Sigma-Aldrich, Cat #G6251) and 50 µM L- Ascorbic acid (Sigma-Aldrich, Cat# A4403). ALP (Beyotime, Cat# C3206), Alizarin Red S(ARS) (Sigma-Aldrich, Cat# A5533; 1% solution in water; pH 4.2) and Von Kossa staining (Servicebio, Cat# GP1054) were performed at 7 days, 21 days and 28 days, respectively. Microscopy images were obtained using a 10 × phase-contrast objective on an Olympus IX73 microscope.

### The proliferation and apoptosis assay

MTT was used to detect the proliferation of BMMs after knockdown or overexpression of BHLHE40. Inoculate treated BMMs (1 × 10^5^ cells/ml) in a 96-well plate. Theer days later, 100ul of complete α-MEM containing 10% MTT buffer (Procell, Cat# PB180519) was added to each well and incubated at 37 °C for 4 h. After removing the supernatant, 100 µl of dimethyl sulfoxide (DMSO) was added to the wells, and a multifunctional microplate reader (PE Enspire) was used to detect the absorbance at a wavelength of 492 nm.

For the apoptosis assay, BMMs with knockdown or overexpressed of BHLHE40 and their controls were gently dissociated with trypsin without EDTA, and apoptosis was detected with an Annexin V-FITC/PI apoptosis kit (MultiSciences, Cat# 70-AP101-100) according to the manufacturer's instructions. CytoFlex S flow cytometer and Cytexpert analysis software (Beckman Coulter) were used for analysis.

### Mice and bone marrow transplantation

*Bhlhe40*^*−/−*^(BHLHE40 KO) mice were a gift from the Collaborative Innovation Center of Model Animal Wuhan University. Targeting of the *Bhlhe40* locus was performed by standard techniques. The BssHII–BssHII genomic fragment of *Bhlhe40* as previously described [47], which contains the promoter and coding region including the ATG in exon 1, was replaced with a Neo cassette. The resulting chimeric mice were backcrossed to C57BL/6 mice more than six to eight times.

For elderly animal models, 1-month-old or 12-month-old male mice (C57BL/6) were sacrificed, and the femurs were isolated and fixed with 4% paraformaldehyde for subsequent experiments.

To establish an osteoporosis mouse model [48], 6-week-old female mice (C57BL/6) were anesthetized with 1–4% isoflurane. A 0.5 cm single midline dorsal incision was made in the lower back through the skin. The connective tissue under the skin was gently freed, the ovary was positioned under the thin muscle layer, and a small incision was made on each side to enter the abdominal cavity of the skin. Expose fallopian tubes and ovaries. The ovaries were identified and placed back into the abdominal cavity (Sham). Ligation was performed around the fallopian tube and small sterile scissors were used to gently cut off the fallopian tube to remove the ovaries. The rest of the fallopian tube was placed back into the abdominal cavity (OVX) and sutured layer by layer.

Since adult osteoclasts are mainly derived from HSCs [6], this allows us to specifically study the effect of gene knockout in osteoclasts on bone mass. Bone marrow transplantation was performed as previously described [49] and grouped as shown in Fig. 4A. In brief, 6-week-old male recipient mice (*Wt* and *Bhlhe40*^*−/−*^) were lethally irradiated (8 Gy). After 4 h, the donor mice (4-week-old *Wt* and *Bhlhe40*^*−/−*^) were sacrificed and the femurs were separated. The bone marrow was collected from the femur by washing 3 times with PBS supplemented with 1% FBS and filtering the cells through a 200 mesh screen. The cells were washed in PBS and resuspended at a concentration of 10^7^ cells/ml. Then, 10^6^ donor cells (100 μl) were injected into the tail vein of each mouse in the recipient group, and the animals were kept for 7 weeks. Peripheral blood was collected (50 µl) to extract DNA for chimerism identification by PCR. The primer sequences used in the assay are listed in Additional file 8: Table S1. No statistical method was used to predetermine the sample size of animal studies. No mice were excluded from analyses except those with unsuccessful transplantation.

### Knockdown and overexpression

To knock down *Bhlhe40, Nfatc1* and *Fos*, a riboFECT CP Transfection Kit (RiboBio, Cat # R10035.7) was used for siRNA transfection for 24 h. The gene sequences used for the target siRNA are listed in Additional file 8: Table S1. The *Bhlhe40* overexpression plasmid was purchased from Shanghai Genechem. Lentivirus packaging and infection were performed as previously described [50]. The plasmids pSPAX2, pMD2.G and overexpression plasmids were cotransfected into 293 T cells to package lentivirus. The supernatant was collected at 48 and 72 h and lentiviruses were concentrated (Millipore, Ultracel-100 regenerated cellulose membrane, 15 mL sample volume, Cat# UFC9100). Lentiviral infection (MOI = 100) of BMMs was performed in the presence of 10 µg/ml polybrene for 24 h. Cells were selected with 2 µg/ml puromycin for 72 h. The knockdown efficiency or overexpression level of *Bhlhe40* was verified by qPCR. The knockdown efficiency levels of *Nfatc1* and *Fos* were verified by qPCR.

### Chromatin immunoprecipitation (ChIP)

The samples were prepared as described previously [51]. BMMs were cultured in 30 ng/ml M-CSF for 3 days and fixed in 1% formaldehyde for 10 min at room temperature. Cells were neutralized with glycine and collected to make cell lysates. Cells were resuspended in sonication buffer at 4 °C for 10 min, and then each sample was sonicated 6 times with 40% AMP for 15 s. After centrifugation at 8000 g for 30 s at 4 °C, chromatin was collected from the supernatant, and 50 μl aliquots of the sample were used to extract the input DNA. After incubating with 1 μl of 1 mg/ml RNase A (Sigma-Aldrich, Cat# R6513) and 2 μl of proteinase K (20 mg/ml) at 65 °C overnight, the input DNA was purified using a TIANGEN Common DNA Product Purification Kit (TIANGEN, Cat# DP204). The chromatin samples were incubated with 1 mg/ml BSA and 40 μl prebalanced protein A/G magnetic beads (MCE, Cat# HY-K0202) for 2 h at 4 °C. After the beads were removed, 1 μg of anti-BHLHE40 antibody was added, incubated overnight and then precipitated with protein A/G magnetic beads. The beads were washed, and DNA was eluted and purified with a TIANGEN Common DNA Product Purification Kit after RNase A treatment. The immunoprecipitated DNA fragments were collected for qPCR amplification. Normal rabbit IgG was used as a negative control. DNA samples were analysed by real-time PCR, and IgG was used for standardization. The nucleotide sequence of the oligonucleotide used in the assay is shown in Additional file 8: Table S1. The antibodies used were summarized in Additional file 9: Table S2.

### RNA extraction and quantitative PCR

TRIzol (Thermo Fisher, Cat# 15596026) was used to extract total RNA from BMMs. The reverse transcriptase kit (Vazyme, Cat# R223) was used to reverse transcribe an aliquot of 200 ng total RNA into cDNA. SYBR green mixture (Vazyme, Cat# Q311) and Monad Real-Time PCR instrument (Monad q225) were used for quantitative PCR. The primers used for specific transcripts are listed in Additional file 8: Table S1. The mRNA level was normalized to that of GAPDH.

### Micro-CT analysis

All mice were euthanized with CO_2_, and the femurs were separated and evaluated by the SkyScan 1176 high-resolution micro-CT imaging system (Bruker, Germany). According to the manufacturer's instructions [49], each femur was scanned separately at 55 kV and 200 μA using a 0.25-mm aluminium filter to obtain an isometric resolution of 7-μm. NRecon (Bruker, Germany) was used to reconstruct the image and CTAn (Bruker, Germany) was used for quantitative analysis. The volume of interest (VOI) contains most of the metaphysis and part of the diaphysis. CTVol software was used (Bruker Micro-CT, Germany) to adjust the 3D model. Trabecular or cortical bone parameters in an area from 0.2 mm to 2.3 mm or 4.0 mm to 5.4 mm below the growth plate of the femur were measured.

### Mechanical testing

The soft tissue was removed from the femur to ensure consistency between the conditions used in the mechanical test. All 8 samples were mechanically tested in the vertical direction. Polymethyl methacrylate rigid base (PMMA, Aladdin, Cat# 9011-14-7) was used to fix both sides of the femur and contact the metal base [52]. The dynamic fatigue testing machine (BOSE ElectroForce 3220 225N) was brought in contact with the PMMA bases on both sides until nominal compression (< 10 N) was observed. Then, the mouse femur was compressed at a speed of 1 mm/s until the applied load dropped significantly (loss of 50% or more). The mechanical test was carried out at room temperature. All test data were collected at 20 Hz.

### Skeletal preparation and staining

As mentioned earlier [53], mice were sacrificed by CO2 and skin and muscles were removed. After fixation in 95% ethanol overnight, remaining samples were transferred to acetone for 48 h. Cartilages were then stained in 0.3% Alcian Blue (Sigma-Aldrich, Cat# A5268) overnight at room temperature. The samples were incubated overnight in 95% ethanol to decolorize and incubated with 1% KOH solution at room temperature for 1 h to remove residual tissue. The KOH solution was removed and incubated with 0.1% ARS solution (Sigma-Aldrich, Cat# A5533) overnight at 4 °C for bone staining. Specimens were incubated in 1% KOH until the excess ARS and tissues were completely cleared, and then stored in glycerol for pictures by Nikon camera.

### Immunohistochemistry and histological analysis

For paraffin sections, femurs were fixed in 4% paraformaldehyde for 48 h, decalcified in 10% EDTA for 28 days, and then embedded in paraffin after procedural dehydration. The tissue was sliced (8 μm) with a Leica RM2235 microtome, deparaffinized, and stained with TRAP (Servicebio, Cat# G1050), Masson (Servicebio, Cat# GP1032), Goldner (Servicebio, Cat# GP1053) and haematoxylin–eosin (H&E).

For frozen sections, femurs were fixed in 4% paraformaldehyde for 6 h and decalcified as previously described [54]. After the samples were washed, they were soaked in PBS containing 30% sucrose at 4 °C for 1–2 days, and embedded in a gel-filled disposable histological plastic mold. We used a Leica CM3050S cryostat to slice the samples (20 μm) at − 25 °C.

For immunohistochemistry, sections were deparaffinized and treated with citrate buffer solution (pH 6.0) 3 times at 95 °C, and treated with 3% H_2_O_2_ for 20 min at room temperature. Samples were then blocked with 5% bovine serum albumin (BSA) at room temperature for 1 h, and incubated with the primary antibody overnight at 4 °C. After the samples were washed, the Polink-2 Plus polymer HRP detection system (ZSGB-BIO, Cat# PV6001) was used for the secondary antibody incubation of the samples, and DAB (ZSGB-BIO, Cat# ZLI-9017) was used for color development. Hematoxylin was used for nuclear staining. After dehydration and fixation, the slices were scanned by the Aperio VERSA 8 (Leica, Germany) scanner.

### Immunofluorescence staining

BMMs and RAW264.7 cells cultured in complete α-MEM containing 30 ng/ml M-CSF and 50 ng/ml RANKL for 0, 2, 4, or 6 days were fixed with 4% paraformaldehyde for 20 min and permeabilized with PBS containing 0.5% Triton-X for 30 min. For anti-BHLHE40 immunofluorescence staining, cells were blocked with PBS containing 5% BSA after fixation and permeabilization, and cells were incubated with BHLHE40 primary antibodies overnight at 4 °C. Then, the cells were incubated with secondary antibodies for 1 h. The images were taken by confocal microscopy (Leica SP8, Germany) and analysed with Leica Application Suite X (LAS X).

For immunofluorescence, before staining with antibodies, the sections were allowed to equilibrate for 30 min at room temperature and rehydrated with PBS at room temperature 3 times for 5 min. After the samples were permeabilized or blocked with PBS containing 0.2% Triton-X 100 or 5% BSA for 1 h, the sections were incubated with the primary antibody overnight at 4 °C. After washing with PBS 3 times for 5 min, sections were incubated with fluorescently labeled secondary antibodies for 1 h. Images were captured with a confocal microscope (Leica SP8) and analysed with LAS X. Sections were also stained with integrin alpha-M (CD11b) (BMMs marker), CTSK (OCs marker) and DAPI (nuclear).

The primary and secondary antibodies used are listed in Additional file 9: Table S2.

### Transcriptomic analysis

BMMs were isolated from *Wt* and *Bhlhe40*^*−/−*^ mice, cultured with 50 ng/ml M-CSF for 3 days, and harvested in a GenCatch TM Total RNA Extraction Kit (Epoch Life Sciences, Cat# 1660050) 48 h after OC differentiation. Two micrograms of RNA was used for cDNA library preparation and sequencing following the manufacturer's instructions (HiSeq 2500, Illumina). HISAT2 [55] was used to map sequencing reads to the mouse genome and SAMtools [56] was used to aggregate tag counts at the gene level allowing only one read per position per length. DESeq2 [57] was used to determine the differentially expressed genes. ClusterProfiler [58] was used to perform gene set enrichment analysis, including GO and KEGG analyses. GOplot [59] was used for the visualization of GO analysis. The RNA-seq data in this study are available in SRA, and the BioProject accession number is PRJNA763091 (http://www.ncbi.nlm.nih.gov/bioproject/763091).

### Bioinformatics analysis

To explore the potential correlation between *Bhlhe40* and OC differentiation, GSE54779 (GPL6246 Affymetrix mouse gene 1.0 ST array) dataset from the Gene Expression Omnibus (GEO) repository at the National Centre of Biotechnology Information website (https://www.ncbi.nlm.nih.gov/geo/) was analysed. This dataset contains three BMM samples treated with RANKL and M-CSF and three BMM samples treated with M-CSF alone. All raw expression data were standardized by R and the Bioconductor (http://www.bioconductor.org) Affymetrix package. The Linear Array Microarray Analysis (Limma) software package was used to identify the differentially expressed genes (DEGs) in BMM samples treated with M-CSF and RANKL compared with samples stimulated with M-CSF alone. Values of *p* < 0.05 and |log2FC|> 0.5 were considered thresholds for identifying DEGs.

In order to determine the correlation of *Bhlhe40* and *Fos* or *Nfatc1*, GSE7158 or GSE51495 from the GEO repository at the National Center for Biotechnology Information website (https://www.ncbi.nlm.nih.gov/geo/) was used for analysis (GPL570 Affymetrix Human Genome U133 Plus 2.0 Array or GPL6104 Illumina humanRef-8 v2.0). GraphPad Prism 7.0 was used to analyse the correlation between *Bhlhe40* and *Fos* or *Nfatc1* expression in each sample.

The BHLHE40 target genes were predicted through the Gene Transcription Regulation Database (GTRD (http://gtrd.biouml.org/)). The binding sites of BHLHE40 were predicted through the JASPAR database (http://jaspar.genereg.net/).

### Statistical analysis

Data were expressed as the mean ± SD. The results were considered statistically significant if the P value was less than 0.05. The random number method was used for random assignment. Researchers blinded to group assignment during the experiment. Individual data points were shown, and the number of samples or images analysed was indicated in the figure and/or legend. The data were analysed using the appropriate Student's t test when comparing two groups or one-way analysis of variance when comparing more than 2 groups. The data points correspond to independent experiments. The variances between the compared groups were similar. All statistical tests were performed using Prism 7.0 software.

## Supplementary Information

**Additional file 1: Figure S1**. OC-specific gene expression of BMMs during osteoclast differentiation (n=3). All data are mean±SD; * P<0.05, **P<0.01, ***P<0.001. by one-way ANOVA followed by Tukey’s post hoc test.

**Additional file 2: Figure S2**. The expression of BHLHE40 in elderly mice (A, B) Representative images of the Immunofluorescence analysis of BHLHE40 (green) and quantitative analysis (n=3) of the bone marrow macrophages (BMMs) between Control (2 months) and Eld (12 months) mice. BMMs was stained with CD11b (red). Nucleus was stained with DAPI (blue) (Scale bar, 150 μm). (C, D) Representative images of the immunofluorescence analysis of BHLHE40 (green) and quantitative analysis (n=3) of the osteoclasts (OCs) between Control (2 months) and Eld (12 months) mice. OCs was stained with CTSK (red). Nucleus was stained with DAPI (blue) (Scale bar, 150 μm). All data are mean±SD; ns P > 0.05, * P < 0.05, * *P < 0.01. by paired Student’s t test.

**Additional file 3: Figure S3**. The effect of Bhlhe40 on the proliferation and apoptosis of BMMs (A) MTT in Si-NC, Si-Bhlhe40, control and Bhlhe40 overexpression BMMs (n=6). (B) Representative flow cytometry plots of cell apoptosis distribution and the percentage of cells in each phase for BMMs with Si-NC, Si-Bhlhe40, control and Bhlhe40 overexpression. All data are mean±SD; ns P > 0.05. by unpaired Student’s t test.

**Additional file 4: Figure S4**. Representative figures of mice genotype identification.

**Additional file 5: Figure S5**. Osteogenic-specific staining between Wt and Bhlhe40-/- at 4-weeks-old (A) Alkaline phosphatase (ALP), Alizarin Red S (ARS), and Von Kossa staining to detect osteoblast differentiation of bone marrow mesenchymal cells (BMSCs) from Wt and Bhlhe40-/- mice (Scale bar, 100 μm). (B, C) Masson and Goldner staining of femurs from Wt and Bhlhe40-/- mice (4 weeks) (Scale bar, 200 um). (D) ARS/Alcian Blue staining of skeleton in Wt and Bhlhe40-/- mice on the third day (P3) after birth (Scale bar, 5 mm). (E, F) Immunohistochemistry of OCN and quantitative analysis (n=3) in femur sections from Wt and Bhlhe40-/- mice (Scale bar,200 μm). All data are mean±SD; ns P > 0.05. by unpaired Student’s t test.

**Additional file 6: Figure S6**. Identification of bone marrow transplantation.

**Additional file 7: Figure S7**. The efficiency detection of Nfatc1 and Fos knock-down (A) RNA expression of Nfatc1 in BMMs between Si-NC and Si- Nfatc1 (n=3). (B) RNA expression of Fos in BMMs between Si-NC and Si-Fos (n=3). All data are mean±SD; * P<0.05, **P<0.01. by unpaired Student’s t test.

**Additional file 8: Table S1**. Primer sequence

**Additional file 9: Table S2**. Antibody source

## Data Availability

The datasets used and/or analysed during the current study are available from the corresponding author on reasonable request. The datasets in this study are available in the following databases: RNA-Seq data: Sequence Read Archive (SRA) PRJNA763091 (http://www.ncbi.nlm.nih.gov/bioproject/763091).

## Abbreviations

BMMs: Bone marrow macrophages
HSCs: Hematopoietic stem cells
OCs: Osteoclasts
M-CSF: Macrophage colony stimulating factor
RANKL: Receptor activator of NF-κB ligand
TRAF6: Tumor necrosis factor receptor–associated factor 6
MAPKactivates: Mitogen-activated protein kinase
NFATc: Nuclear factor of activated T cells
AP-1: Activator protein-1
Mic1: Macrophage inhibitory cytokine-1
FBS: Foetal bovine serum
H&E: Haematoxylin–eosin
GEO: Gene Expression Omnibus
VOI: Volume of interest
BSA: Bovine serum albumin
GTRD: Gene Transcription Regulation Database
OVX: Ovariectomization
ARS: Alizarin Red S
BMSCs: Bone marrow mesenchymal cells
OCN: Osteocalcin
DEGs: Differentially expressed genes
MIOP: Malignant infantile osteopetrosis
BV/TV: Bone volume fraction
Tb. N: Number of trabeculae
Tb. Th: Trabecular bone thickness
Tb. Sp: Trabecular bone separation
BMD: Bone mineral density
Ct. Th: Cortical bone thickness
BS/TV: Bone surface area bone volume ratio
SMI: Structural model index
Tb. Pf: Trabecular bone model factor
