# Supplementary figures and images for: BHLHE40 promotes osteoclastogenesis and abnormal bone resorption via c-Fos/NFATc1
Source: Cell Biosci. 2022 May 26;12:70. doi: 10.1186/s13578-022-00813-7 (PMC9134610; doi:10.1186/s13578-022-00813-7)

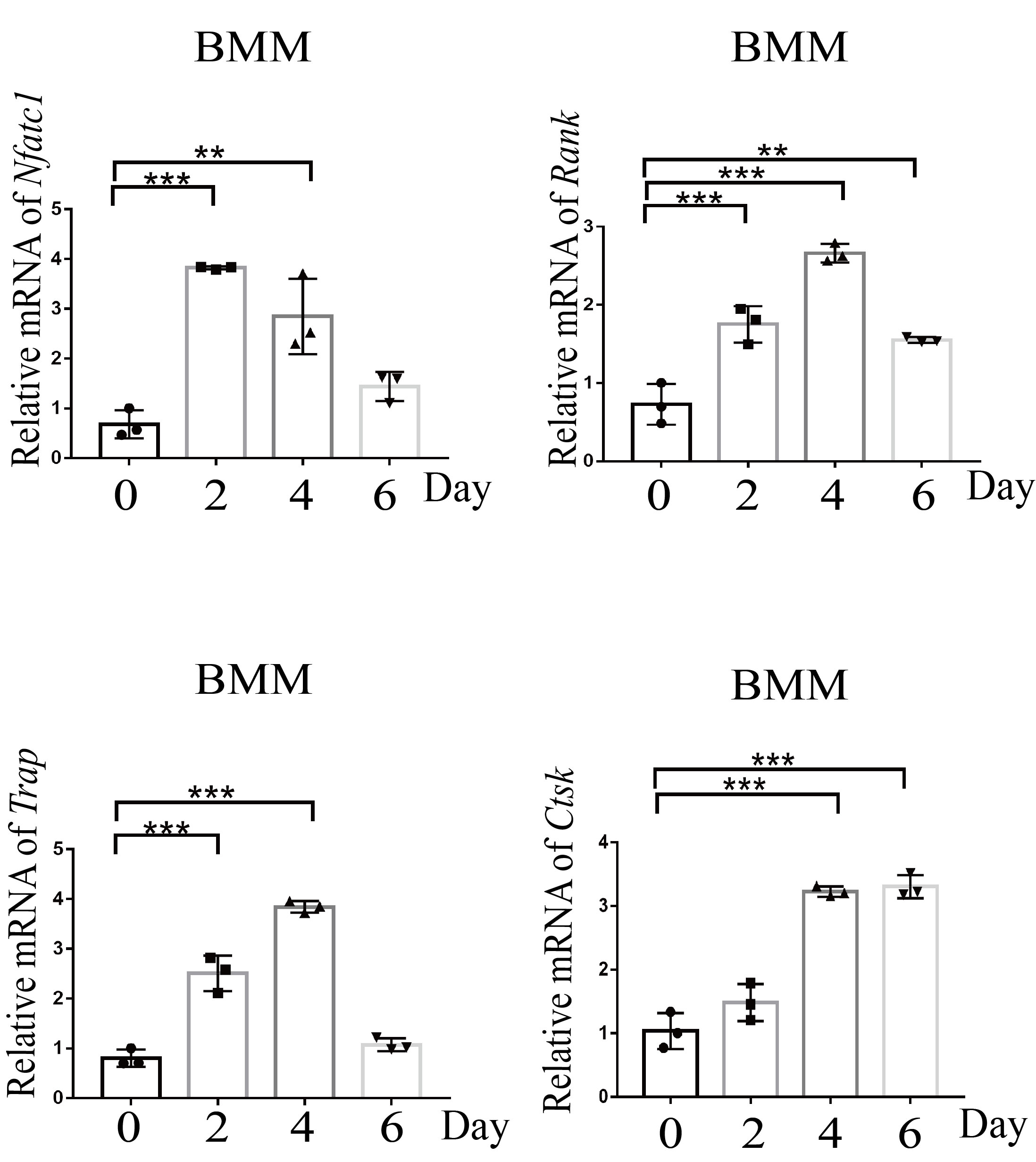

Supplement: Supplementary file 1 — Additional file 1: Figure S1. OC-specific gene expression of BMMs during osteoclast differentiation (n=3). All data are mean±SD; * P<0.05, **P<0.01, ***P<0.001. by one-way ANOVA followed by Tukey’s post hoc test. [file 13578_2022_813_MOESM1_ESM.jpg]

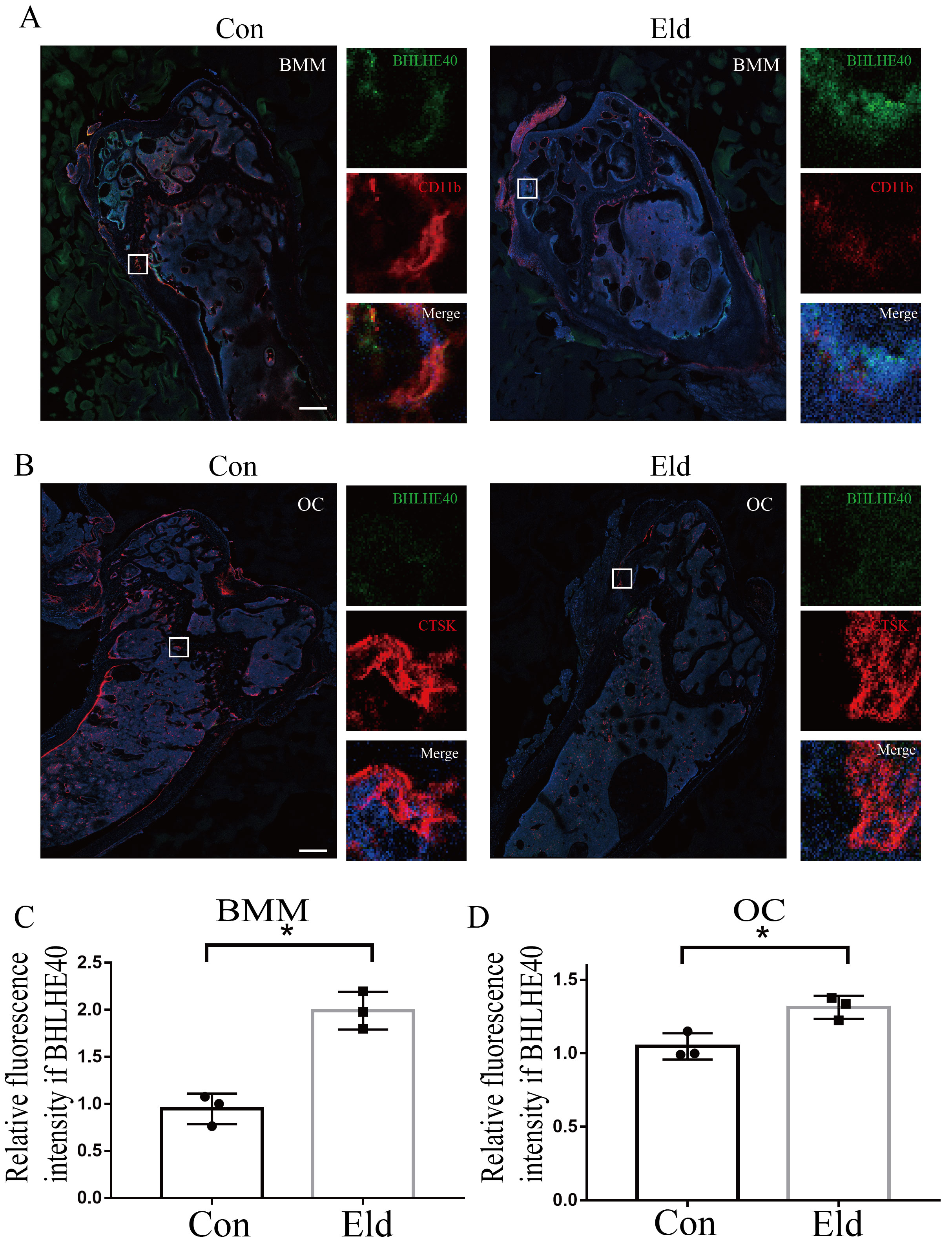

Supplement: Supplementary file 2 — Additional file 2: Figure S2. The expression of BHLHE40 in elderly mice (A, B) Representative images of the Immunofluorescence analysis of BHLHE40 (green) and quantitative analysis (n=3) of the bone marrow macrophages (BMMs) between Control (2 months) and Eld (12 months) mice. BMMs was stained with CD11b (red). Nucleus was stained with DAPI (blue) (Scale bar, 150 μm). (C, D) Representative images of the immunofluorescence analysis of BHLHE40 (green) and quantitative analysis (n=3) of the osteoclasts (OCs) between Control (2 months) and Eld (12 months) mice. OCs was stained with CTSK (red). Nucleus was stained with DAPI (blue) (Scale bar, 150 μm). All data are mean±SD; ns P > 0.05, * P < 0.05, * *P < 0.01. by paired Student’s t test. [file 13578_2022_813_MOESM2_ESM.jpg]

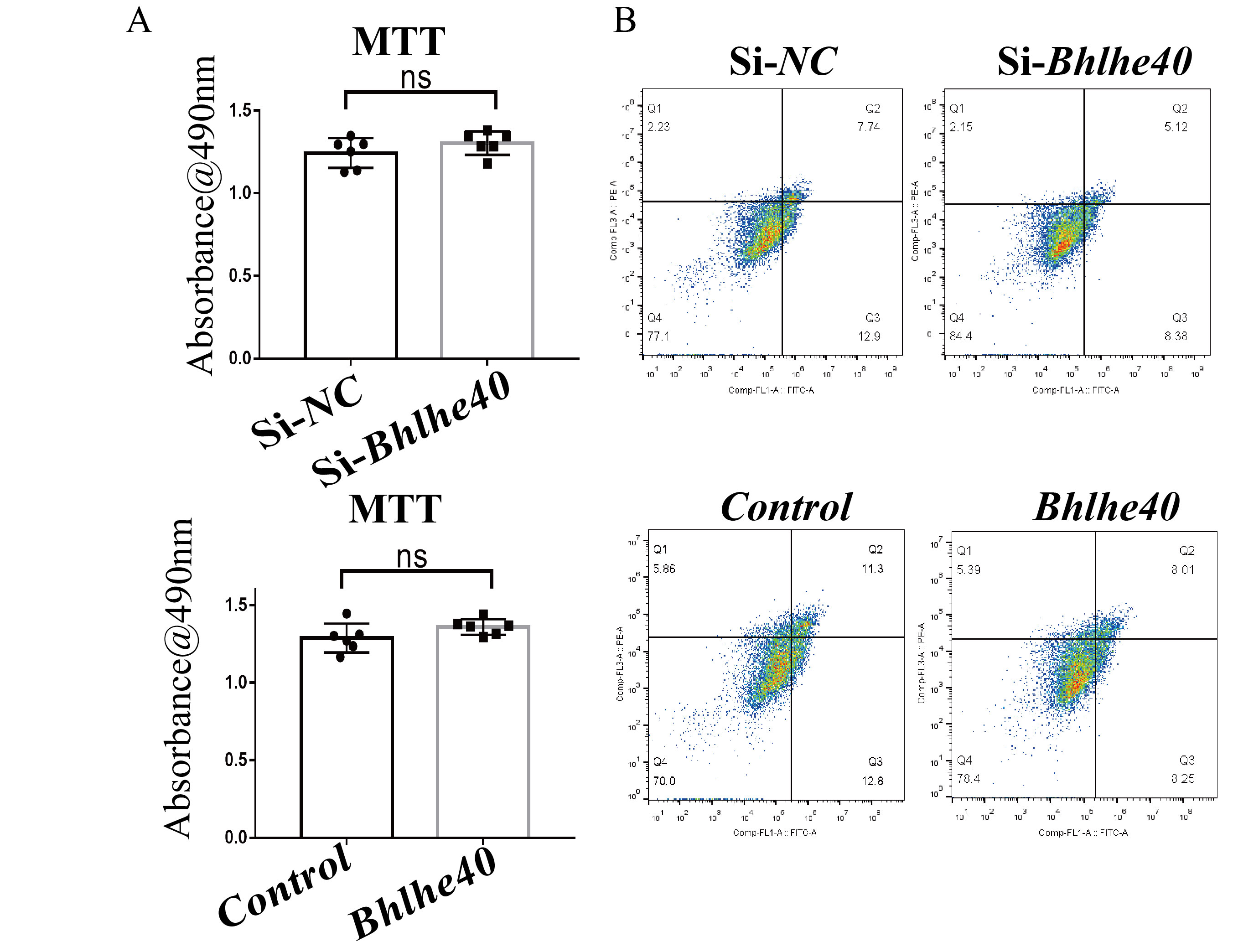

Supplement: Supplementary file 3 — Additional file 3: Figure S3. The effect of Bhlhe40 on the proliferation and apoptosis of BMMs (A) MTT in Si-NC, Si-Bhlhe40, control and Bhlhe40 overexpression BMMs (n=6). (B) Representative flow cytometry plots of cell apoptosis distribution and the percentage of cells in each phase for BMMs with Si-NC, Si-Bhlhe40, control and Bhlhe40 overexpression. All data are mean±SD; ns P > 0.05. by unpaired Student’s t test. [file 13578_2022_813_MOESM3_ESM.jpg]

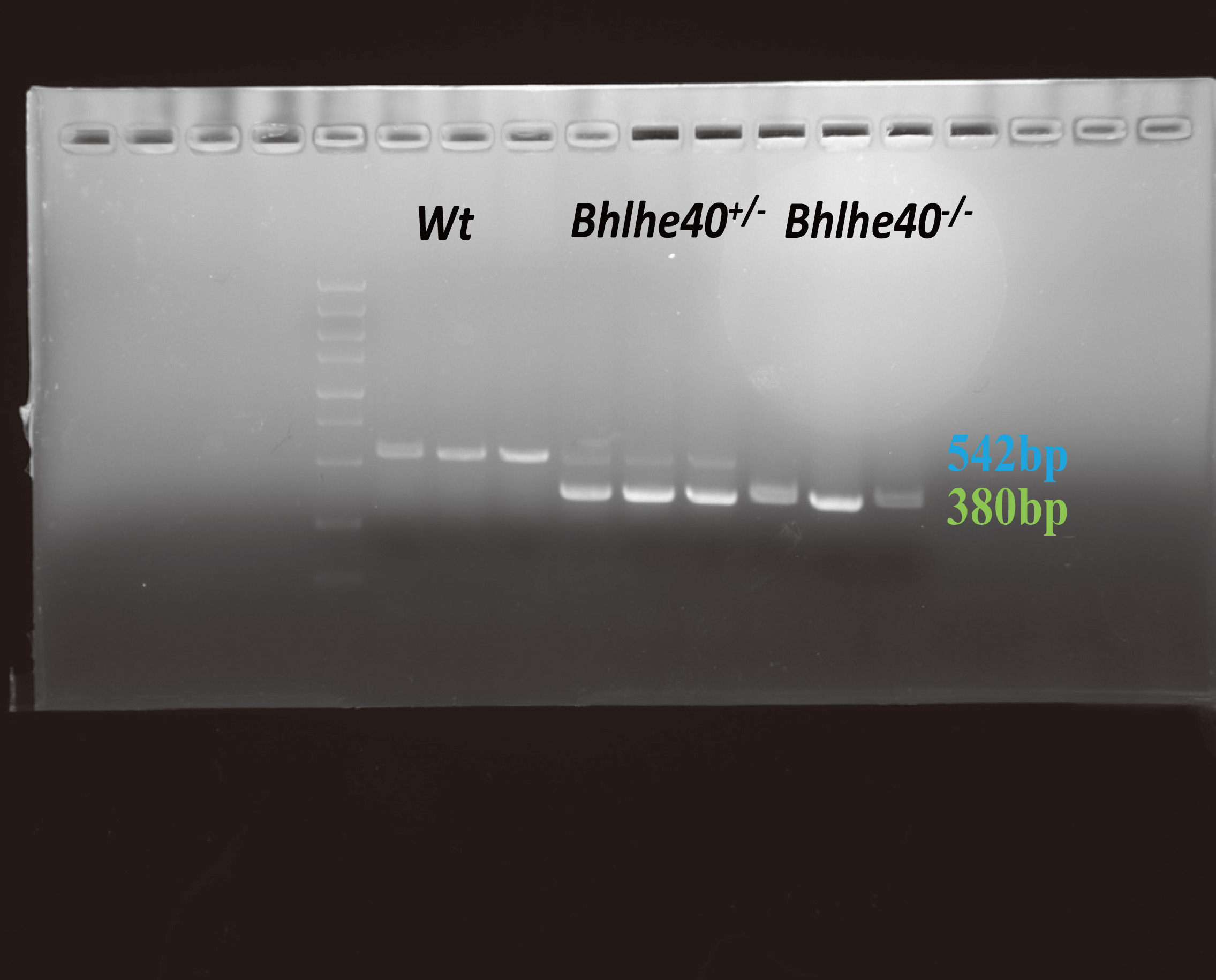

Supplement: Supplementary file 4 — Additional file 4: Figure S4. Representative figures of mice genotype identification. [file 13578_2022_813_MOESM4_ESM.jpg]

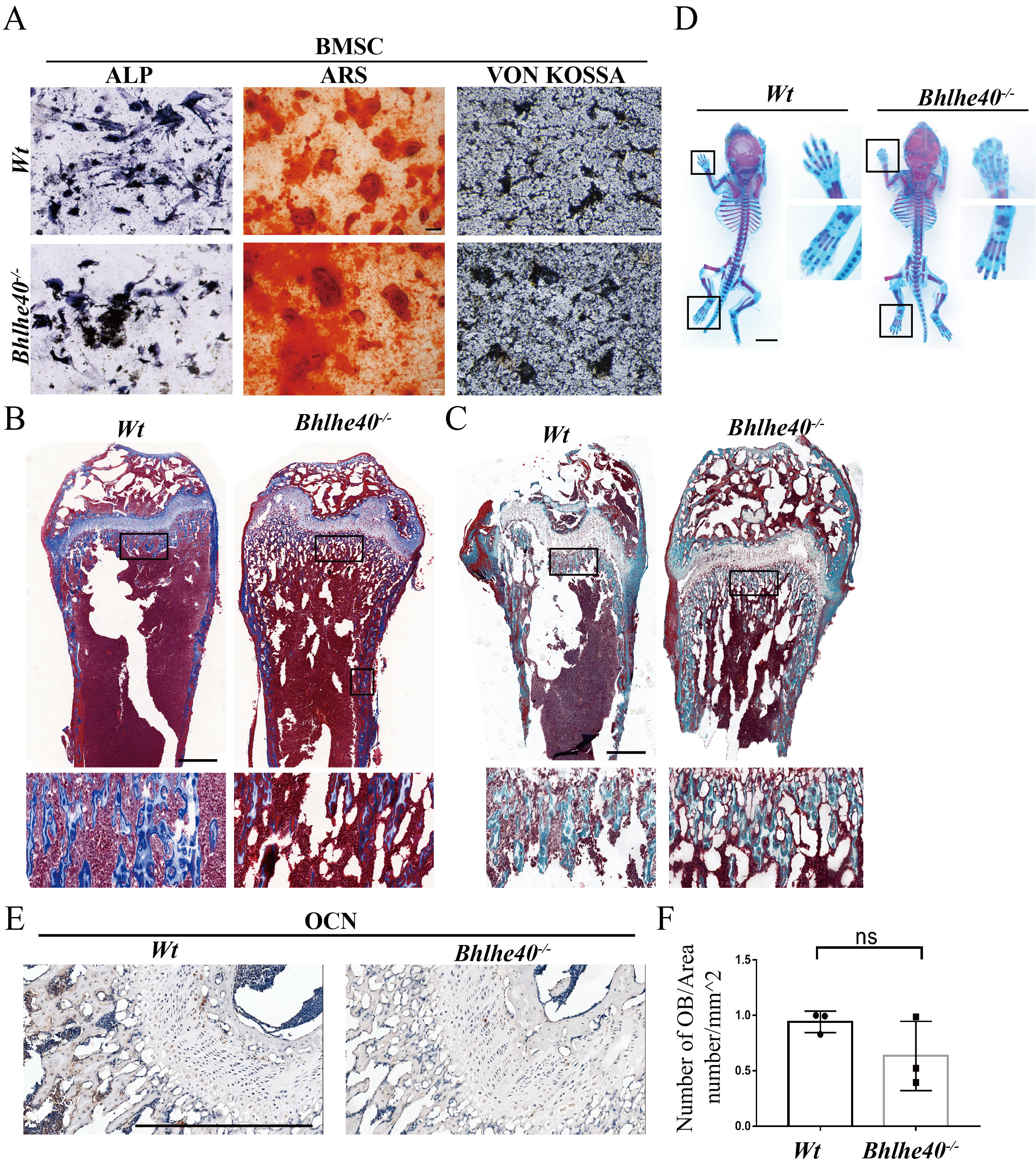

Supplement: Supplementary file 5 — Additional file 5: Figure S5. Osteogenic-specific staining between Wt and Bhlhe40-/- at 4-weeks-old (A) Alkaline phosphatase (ALP), Alizarin Red S (ARS), and Von Kossa staining to detect osteoblast differentiation of bone marrow mesenchymal cells (BMSCs) from Wt and Bhlhe40-/- mice (Scale bar, 100 μm). (B, C) Masson and Goldner staining of femurs from Wt and Bhlhe40-/- mice (4 weeks) (Scale bar, 200 um). (D) ARS/Alcian Blue staining of skeleton in Wt and Bhlhe40-/- mice on the third day (P3) after birth (Scale bar, 5 mm). (E, F) Immunohistochemistry of OCN and quantitative analysis (n=3) in femur sections from Wt and Bhlhe40-/- mice (Scale bar,200 μm). All data are mean±SD; ns P > 0.05. by unpaired Student’s t test. [file 13578_2022_813_MOESM5_ESM.jpg]

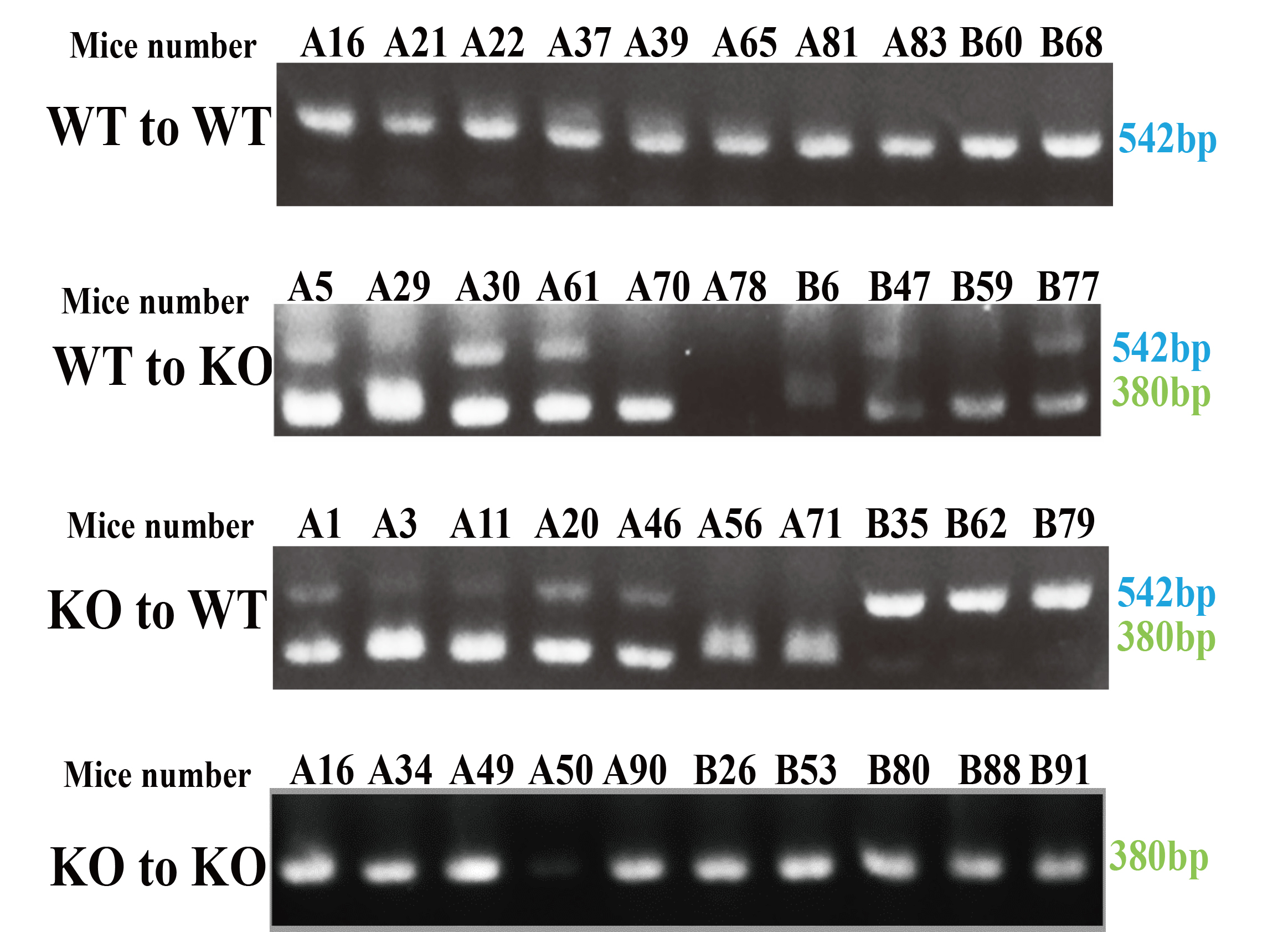

Supplement: Supplementary file 6 — Additional file 6: Figure S6. Identification of bone marrow transplantation. [file 13578_2022_813_MOESM6_ESM.jpg]

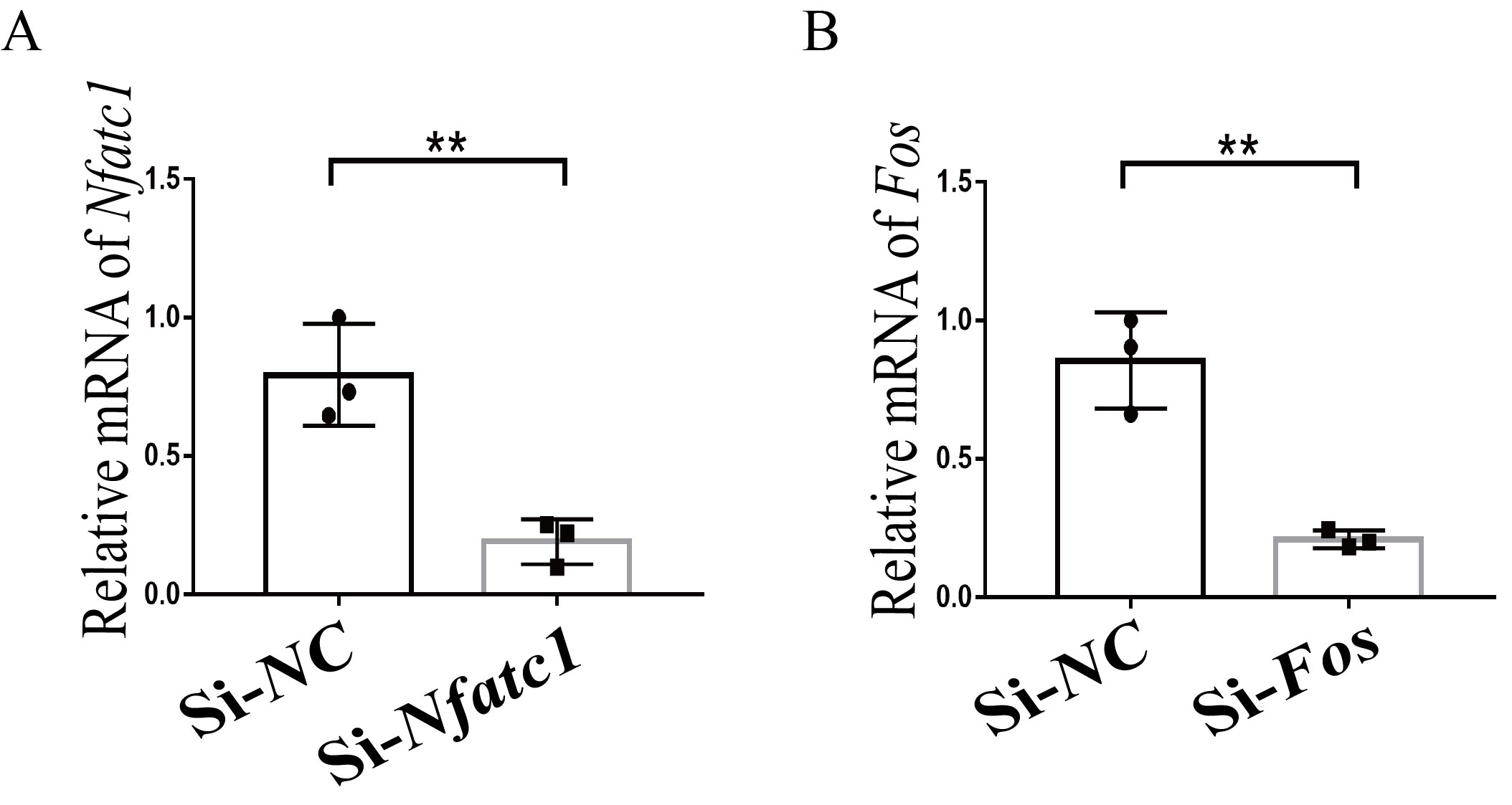

Supplement: Supplementary file 7 — Additional file 7: Figure S7. The efficiency detection of Nfatc1 and Fos knock-down (A) RNA expression of Nfatc1 in BMMs between Si-NC and Si- Nfatc1 (n=3). (B) RNA expression of Fos in BMMs between Si-NC and Si-Fos (n=3). All data are mean±SD; * P<0.05, **P<0.01. by unpaired Student’s t test. [file 13578_2022_813_MOESM7_ESM.jpg]
